# NPC1-dependent alterations in K_V_2.1–Ca_V_1.2 nanodomains drive neuronal death in models of Niemann-Pick Type C disease

**DOI:** 10.1038/s41467-023-39937-w

**Published:** 2023-07-28

**Authors:** Maria Casas, Karl D. Murray, Keiko Hino, Nicholas C. Vierra, Sergi Simó, James S. Trimmer, Rose E. Dixon, Eamonn J. Dickson

**Affiliations:** 1 Department of Physiology and Membrane Biology, School of Medicine, University of California, Davis, CA USA; 2 Department of Psychiatry & Behavioral Sciences, School of Medicine, University of California, Davis, CA USA; 3 Department of Cell Biology and Human Anatomy, School of Medicine, University of California, Davis, CA USA

**Keywords:** Ion channels in the nervous system, Mechanisms of disease, Cell death in the nervous system

## Abstract

Lysosomes communicate through cholesterol transfer at endoplasmic reticulum (ER) contact sites. At these sites, the Niemann Pick C1 cholesterol transporter (NPC1) facilitates the removal of cholesterol from lysosomes, which is then transferred to the ER for distribution to other cell membranes. Mutations in NPC1 result in cholesterol buildup within lysosomes, leading to Niemann-Pick Type C (NPC) disease, a progressive and fatal neurodegenerative disorder. The molecular mechanisms connecting NPC1 loss to NPC-associated neuropathology remain unknown. Here we show both in vitro and in an animal model of NPC disease that the loss of NPC1 function alters the distribution and activity of voltage-gated calcium channels (Ca_V_). Underlying alterations in calcium channel localization and function are K_V_2.1 channels whose interactions drive calcium channel clustering to enhance calcium entry and fuel neurotoxic elevations in mitochondrial calcium. Targeted disruption of K_V_2–Ca_V_ interactions rescues aberrant Ca_V_1.2 clustering, elevated mitochondrial calcium, and neurotoxicity in vitro. Our findings provide evidence that NPC is a nanostructural ion channel clustering disease, characterized by altered distribution and activity of ion channels at membrane contacts, which contribute to neurodegeneration.

## Abstract (web summary)

The molecular mechanisms connecting Niemann Pick C1 cholesterol transporter (NPC1) loss to Niemann-Pick Type C neuropathology remain unknown. Here, the authors demonstrate that loss of NPC1 function alters the nanoscale distribution and function of ion channels to promote abnormal calcium entry, mitochondrial dysfunction, and neurotoxicity.

## Introduction

Cholesterol, an essential sterol, is particularly abundant in the brain^1,2^ where it fulfils important biophysical and signaling functions that affect ion permeability, cellular signaling, and general programs of transcription (for general review see refs. ^1,3–7^). Given the importance of maintaining cholesterol homeostasis for controlled neuronal function, it is not surprising that mutations that disrupt cholesterol production or transport result in severe abnormalities of the central nervous system (CNS) and neurodegeneration^8,9^.

In the brain, all cholesterol is made locally^10^ with neurons drawing their homeostatic requirements from two sources (i) de novo synthesis in the Endoplasmic Reticulum (ER), and (ii) uptake of the externally derived cholesterol-conjugated lipoproteins. External cholesterol is imported into neurons in a receptor-dependent manner and trafficked to late endosomes /lysosomes^11^. At these vesicular structures, the Niemann Pick type C (NPC) proteins are key players in mobilizing cholesterol to the ER and other cellular membranes^12,13^. Specifically, following liberation by acidic lipases in the lysosome lumen, cholesterol binds NPC2, which subsequently interacts with the transmembrane NPC1 cholesterol transporter to facilitate the egress of cholesterol across the lysosome membrane to its cytoplasmic leaflet before subsequent transfer to the ER at lysosome–ER Membrane Contact Sites (MCSs). The importance of the NPC1 protein is clear; physiologically, it is a key gatekeeper for cholesterol homeostasis and in doing so tunes mTORC1 activity, lipid transfer at membrane contact sites, and Ca^2+^ signaling^14–17^. Pathophysiologically, missense mutations in NPC1 lead to the progressive neurodegenerative disorder NPC disease. This terminal condition has no cure and is characterized by the progressive neurodegeneration of several brain regions and a host of devastating symptoms including seizures, psychiatric problems, and dementia. Despite its ubiquitous expression, key role in cholesterol transport, and fundamental requirement for human health, the molecular mechanism(s) linking loss of NPC1 function to neurodegeneration are unknown.

A key mechanism through which the lysosome communicates instructions to other organelles is through physical membrane contacts. These intracellular synapses are intimate sites (~10–30 nm) of membrane contact between two or more organelles^18–22^ that use lipids and Ca^2+^ as “currency” to communicate information. A key signaling lipid that is transferred at ER–lysosome MCSs is cholesterol. The NPC1 protein not only influences cholesterol transport at lysosome–ER MCSs but is also a master regulator of other MCSs within mammalian cells^14,16,17,23,24^. Loss of function or disease mutations in NPC1 cholesterol transporter results in the remodeling of ER–lysosome^14,15^, ER–Golgi^16^ and ER–Mitochondria^17^ MCSs to influence mTORC1 signaling, anterograde trafficking to the Plasma Membrane (PM), and mitochondrial Ca^2+^, respectively. That said, there is little information as to how lysosomal cholesterol transport alters the molecular choreography of neuronal ER–PM junctions. This is important because (i) 40–90% of cellular cholesterol is found in the PM^7,25^, therefore disruption of NPC1-mediated cholesterol homeostasis would be expected to result in modification of ER–PM MCSs, and (ii) ER–PM MCSs are critical platforms for regulating lipid and Ca^2+^ homeostasis in neurons^2,26–31^. Despite such importance, we do not know how the loss of cholesterol transport from the lysosome alters ER–PM MCSs, which proteins within these contacts have altered distribution/activity, and the cellular consequences of such changes.

In neurons, a key regulator of ER–PM MCSs is the voltage-gated potassium channel K_V_2.1. Gating of this voltage-dependent ion channel provides an inhibitory brake on excitability^32,33^. However, over the past few years it has been appreciated that a subset of non-conducting K_V_2 ion channels perform a physical function by generating ER–PM junctions through an association between their C-terminus and ER-localized VAP proteins in a phosphorylation-dependent manner^26,34,35^. Moreover, at ER–PM MCSs, K_V_2 channels physically interact with and cluster Ca^2+^ signaling proteins like voltage-gated L-type Ca^2+^ channels (Ca_V_1)^2,31,36^. Ca_V_1 channels are key neuronal Ca^2+^ signaling proteins which serve as a principal route of Ca^2+^ entry at depolarized membrane potentials. Such biophysical properties give Ca_V_1 a prominent role in neuronal excitability and gene expression, which are crucial for neuronal signaling and synaptic plasticity^37^. Moreover, several neurodegenerative diseases are characterized by excessive Ca^2+^ influx through Ca_V_1 channels^38,39^ with their targeted knockdown or inhibition being neuroprotective^40–42^. While K_V_2-mediated targeting and regulation of Ca_V_1 channels at ER–PM MCSs serves a physiologically important role in neurons, there is no information on how this form of regulation may be altered in disease, including how lysosomal cholesterol may regulate K_V_2 clustering to tune excitability and Ca^2+^ signaling at ER–PM MCSs to impact neurodegeneration in NPC disease.

In this current study, we report that loss of NPC1 function results in phosphorylation-dependent increases in K_V_2.1 clustering at the PM. We show that enhanced Kv2.1-containing ER-PM MCSs are enriched in SERCA, Ca_V_1.2, and RyR resulting in enhanced Ca^2+^ signaling at K_V_2.1 associated ER–PM nanodomains. In addition to remodeling of ER–PM MCSs, we also show that Ca^2+^ handling proteins at ER–Mitochondrial MCSs are modified following loss of NPC1 function. Collaboratively, this molecular transformation of MCSs leads to deviant elevations in mitochondrial Ca^2+^ and neurotoxicity. Importantly, we demonstrate that uncoupling upstream K_V_2–Ca_V_1 interactions rescues mitochondrial Ca^2+^ and neurotoxicity and thus represents a potential therapeutic target in NPC1 disease. Collectively, these data demonstrate that NPC is a nanostructural ion channel clustering disease with altered ion channel distribution/activity at MCSs contributing to neurodegeneration.

## Results

### Loss of NPC1 function increases voltage-dependent Ca^2+^ entry and Ca_V_1 channel clustering in isolated neurons

**Fig. 1:**
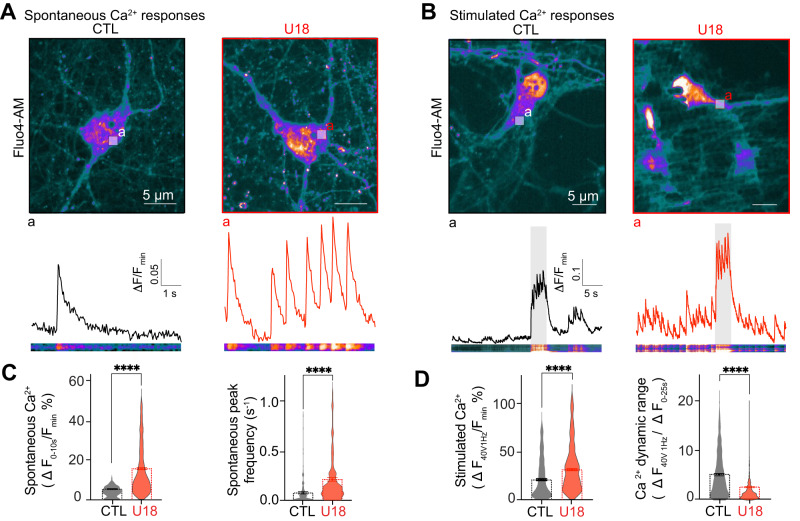
Loss of NPC1 function increases voltage-dependent Ca^2+^ entry. **A** *Top*, representative images of live CTL (black) and U18-treated (red) neurons loaded with fluo-4. *Bottom*, intensity time series and kymographs of spontaneous activity taken from the white region of interest (*a*) in both images. **B** *Top*, representative images of live CTL (black) and U18-treated (red) neurons loaded with fluo-4. *Bottom*, intensity time series and kymographs of stimulated activity taken from the white region of interest (*a*) in both images. Electrical stimulation (gray bar) was performed from seconds 26 to 31 at 40 V 1 Hz. **C** Quantification of spontaneous Ca^2+^ peak amplitude and frequency in CTL (black) and U18-treated (red) neurons. n = 108 (CTL) and n = 148 (U18) neurons, and n = 138 (CTL) and n = 683 (U18) peaks were analyzed over 2 independent isolations. **D** Quantification of Ca^2+^ peak amplitude and Ca^2+^ dynamic range (relative change in Fluo-4 intensity between basal activity and following stimulation) in CTL (black) vs U18-treated (red) neurons. *N* = 457–406 (CTL) and *n* = 656–707 (U18) Ca^2+^ peaks, respectively, over 2 independent isolations. All error bars represent SEM. Statistical significance was calculated using Mann–Whitney *t* test (two-tailed) in (**C**) and (**D**). ****P < 0.0001. CTL is control and U18 is U18666A.

The opening of voltage-gated Ca^2+^ channels (Ca_V_) permits movement of Ca^2+^ down its concentration gradient to increase intracellular Ca^2+^ levels and represents a major mechanism through which neurons translate electrical signals into biochemical ones. Elevations in Ca^2+^ are tightly regulated in mammalian neurons^43,44^ with dysregulation linked to several neuropathologies^45–51^. Recently, we have reported that NPC1 inhibition, NPC1 mutations (NPC1^I1061T^) or NPC1 knockout (NPC1^−/−^) neurons are hyperexcitable, firing significantly more Action Potentials (APs) compared to age and sex-matched Wild-Type (WT) littermates^52^. Given the importance of the membrane potential for controlling Ca^2+^ entry through Ca_V_ channels^53^, we wanted to test if NPC1 loss of function alters voltage-dependent Ca^2+^ activity. To test this hypothesis, we incubated cortical neurons overnight using a specific pharmacological inhibitor of NPC1^54,55^ (U18666A; referred to as U18 hereafter), which increases spontaneous and stimulated voltage responses as well as internal cholesterol accumulation (Figure S1A)^52^, and loaded cells with the cytosolic Ca^2+^ dye, Fluo-4. Quantitative analysis from Fluo-4 time-series experiments revealed that U18 treatment increased the amplitude and frequency of both spontaneous and stimulated (40 V, 1 Hz) elevations in Fluo-4 intensity relative to controls (Fig. 1A–D). Interestingly, the relative change in Fluo-4 intensity between basal activity and following stimulation was decreased compared to control (ΔF_40V 1Hz_/ΔF_0-25s_ in Fig. 1D), suggesting a narrower dynamic range for Ca^2+^ signaling in NPC. Furthermore, preventing AP generation by treatment with the voltage-gated sodium channel blocker Tetrodotoxin (TTX) not only abrogated any NPC1-driven increase in basal and electrically evoked Fluo-4 intensities, but also reduced both spontaneous and stimulated cytosolic Ca^2+^ amplitude elevations compared to control neurons (Fig. S1B).

**Fig. 2:**
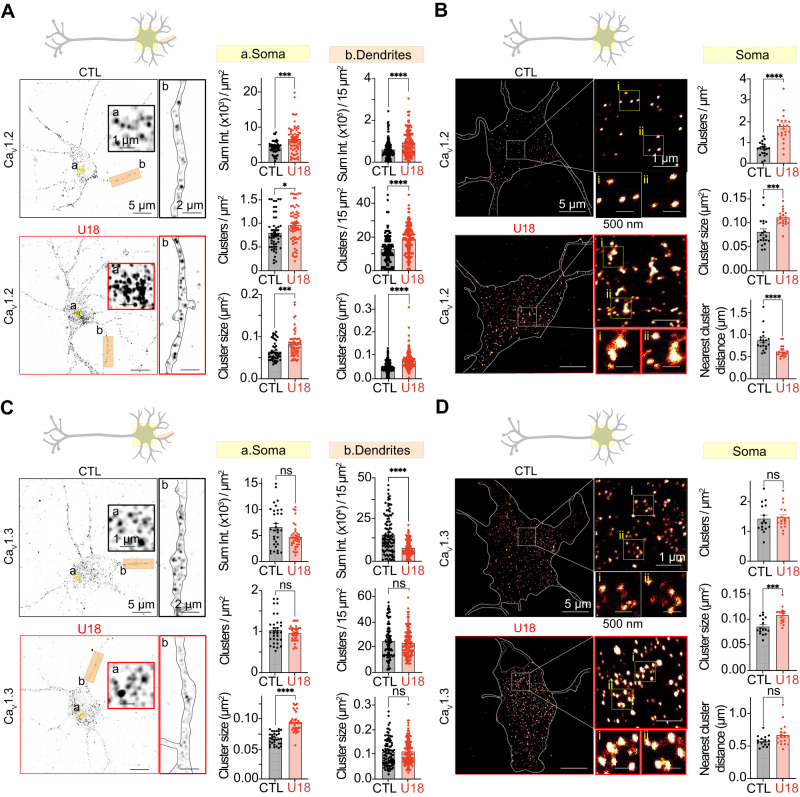
Loss of NPC1 function alters voltage-dependent Ca_V_1 channel distribution. **A** Left: Representative super-resolution Airyscan images taken at a focal plane near the plasma membrane (PM) from CTL (black) and U18-treated (red) neurons fixed and immunolabeled for Ca_V_1.2. *Right*, quantification of PM Ca_V_1.2 total intensity, cluster density and cluster size of CTL (black) and U18-treated (red) neurons in the soma (left, yellow) and dendrite (right, orange) regions. *N* = 46–48 (CTL) and *n* = 58–59 (U18) neurons, and *n* = 145 (CTL) and *n* = 161 (U18) dendrites were analyzed across 5 independent isolations. **B** *Left*, representative super-resolution TIRF localization maps of CTL (black) and U18-treated (red) neurons fixed and immunolabeled for Ca_V_1.2. *Right*, quantification of PM Ca_V_1.2 cluster density, cluster size, and nearest cluster distance of CTL (black) and U18-treated (red) neurons in the soma region. *N* = 20 (CTL) and *n* = 21 (U18) neurons were analyzed across 3 independent isolations. **C** Same as (**A**) only neurons fixed and immunolabeled for Ca_V_1.3. *N* = 32 (CTL) and *n* = 38 (U18) neurons, and *n* = 104 (CTL) and *n* = 123 (U18) dendrites were analyzed across 3 independent isolations. **D** Same as B, only immunolabeled for Ca_V_1.3. *N* = 16 (CTL) and *n* = 18 (U18) neurons were analyzed across 2 independent isolations. All error bars represent SEM. Statistical significance was calculated using Mann–Whitney (two-tailed) and Unpaired *t* tests (two-tailed) in (**A**)–(**D**). ns: not significant; *P < 0.05; **P < 0.01; ***P < 0.001; ****P < 0.0001. CTL is control and U18 is U18666A.

Increased frequency of voltage-dependent Ca^2+^ entry is likely driven by the hyperexcitability phenotype in NPC disease^52^. However, increases in the amplitude of Ca^2+^ events suggested to us that in addition to enhanced hyperexcitability, Ca_V_ channel expression and/or distribution may be altered following the loss of NPC1 function. A salient feature of Ca_V_1 channels in neurons, cardiac myocytes, and smooth muscle is that their activity is regulated by clustering (for review see ref. ^56^), such that adjacent channels can functionally interact with one another permitting cooperative gating^57–61^. As a result, the activity of physically interacting channels in a cluster is driven by the highest open probability channel, thus enhanced channel clustering facilitates the amplification of Ca^2+^ currents. To test if NPC1 loss of function alters Ca_V_ distribution we treated cortical with U18 before fixing and immunolabeling for Ca_V_1.2 and performing super-resolution AiryScan or single molecule localization TIRF microscopy (super-res_TIRF_). Quantification of total intensity, cluster density, and cluster size from AiryScan confocal regions close to the PM demonstrated that U18-treatment enhanced Ca_V_1.2 clustering in both the soma and dendrites (Fig. 2A). Analysis of super-res_TIRF_ localization maps (resolution of 30 nm^62^) further corroborated our AiryScan analysis that U18-treatment increased the size and number of PM Ca_V_1.2 clusters (Fig. 2B). Moreover, nearest neighbor cluster distance analysis revealed that Ca_V_1.2 clusters are in closer spatial proximity following NPC1 loss of function (Fig. 2B).

In addition to Ca_V_1.2, cortical neurons have other L- and P/Q-type Ca_V_ channels that contribute to voltage-dependent Ca^2+^ entry. To test if these Ca_V_ channel isoforms are also altered, we immunolabeled cortical neurons with antibodies against Ca_V_1.3 or Ca_V_2.1 and quantified their distributions at the PM using super-resolution microscopy. Ca_V_1.3 cluster sizes were significantly increased upon U18 treatment while the total fluorescence intensity, number of clusters and spatial proximity between adjacent channels was unaltered in the somatic region of neurons (Fig. 2C, D). Unlike the soma, Ca_V_1.3 cluster size and density in dendrite regions remained unchanged while total fluorescence intensity significantly decreased (Fig. 2C). In contrast to Ca_V_1.2 and Ca_V_1.3, quantification of Ca_V_2.1 revealed a significant reduction in cluster size and intensity with no significant difference in cluster number at the soma, while Cav2.1 clustering in dendrites remained unaltered by U18 (Fig. S2). Collectively, these data provide evidence that loss of NPC1 function facilitates clear and broad alterations in Ca_V_1 channel distribution with significant increases in the size of Ca_V_1.2 and Ca_V_1.3 channel clusters in the cell soma.

### K_V_2.1 clustering is enhanced following loss of NPC function in-vitro and in animal models of NPC disease

Having demonstrated that the NPC1 cholesterol transporter influences the distribution of PM Ca_V_ channels, we next wanted to determine the molecular mechanism that increases somatic Ca_V_1.2 and Ca_V_1.3 channel clustering. To begin, we tested if a simple increase in Ca_V_1.2 protein levels could account for increases in channel clustering. To that end, we extracted protein from cortical neurons cultured for 6-8 Days In Vitro (DIV) and found that total Ca_V_1.2 protein levels were similar between control and U18-treated neurons (Fig. S3A) suggesting a simple increase in protein expression is unlikely to underlie increases in PM Ca_V_ channel distribution.

**Fig. 3:**
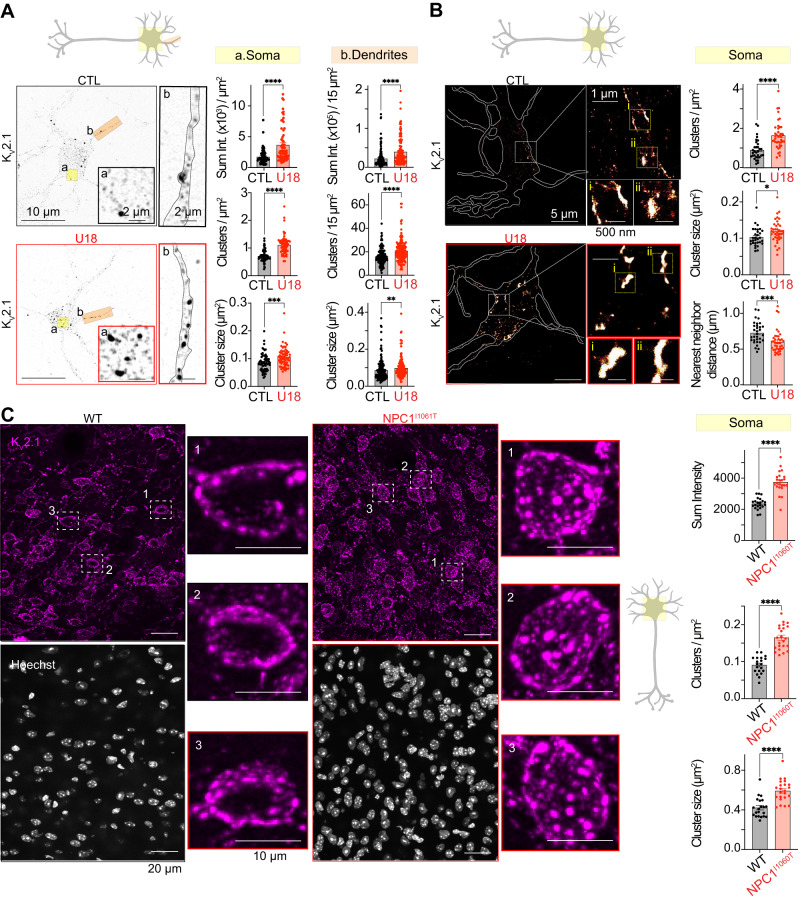
Neurons lacking functional NPC1 have altered distribution of plasma membrane K_V_2.1. **A** *Left*, representative super-resolution Airyscan images taken at a focal plane near the PM of CTL (black) and U18-treated (red) neurons fixed and immunolabeled for K_V_2.1. *Right*, quantification of PM K_v_2.1 total intensity, cluster density and cluster size of CTL (black) and U18-treated (red) neurons in the soma (left, yellow) and dendrite (right, orange) regions. *N* = 61 (CTL) and *n* = 64 (U18) neurons and *n* = 143 (CTL) and *n* = 162 (U18) dendrites were analyzed across 5 independent isolations. **B** *Left*, representative super-resolution TIRF images of CTL (black) and U18-treated (red) neurons fixed and immunolabeled for K_V_2.1. *Right*, quantification of PM K_V_2.1 cluster density, cluster size and nearest cluster distance of CTL (black) and U18-treated (red) neurons in the soma region. N = 32 (CTL) and n = 40 (U18) neurons were analyzed across 5 independent isolations. **C** *Left*, representative maximum intensity projections from Wild-Type (WT) (black) and NPC1^I1061T^ (red) cortical sagittal sections stained with Hoechst and immunolabeled for K_V_2.1. *Right*, quantification of K_v_2.1 total intensity, cluster size and cluster density of WT (black) and NPC1^I1061T^ (red) neurons in the soma region. N = 20 − 22 (WT) and n = 23 (U18) neurons were analyzed across 2 pairs of animals. All error bars represent SEM. Statistical significance was calculated using Mann-Whitney (two-tail) and unpaired *t* test (two-tail) in (**A**)–(**C**); ns: not significant; *P < 0.05; **P < 0.01; ***P < 0.001; ****P < 0.0001. CTL is control, WT is wild-type, and U18 is U18666A.

Recently, voltage-gated K_V_2.1 potassium channels have been demonstrated to organize neuronal Ca_V_1 channels, impacting their distribution and activity in specific somatic microdomains^2,31^. Based on our observations that somatic Ca_V_1.2 and Ca_V_1.3 channel clusters are increased in NPC disease conditions, we tested if K_V_2.1 channels also had altered distribution. Confocal and super-res_TIRF_ analysis from fixed cortical neurons immunolabeled for K_V_2.1 revealed that the total fluorescence intensity, number of clusters, and cluster size were all increased in the PM from somatic and dendritic regions of U18-treated neurons relative to control (Fig. 3A). Additionally, in-depth analysis of super-res_TIRF_ maps determined that not only are K_V_2.1 channel clusters larger, but they are closer together with U18 decreasing nearest neighbor distance between adjacent K_V_2.1 channel clusters (Fig. 3B). Like Ca_V_1.2 channels, total levels of K_V_2.1 expression remained constant between control and U18 conditions (Fig. S3A).

**Fig. 4:**
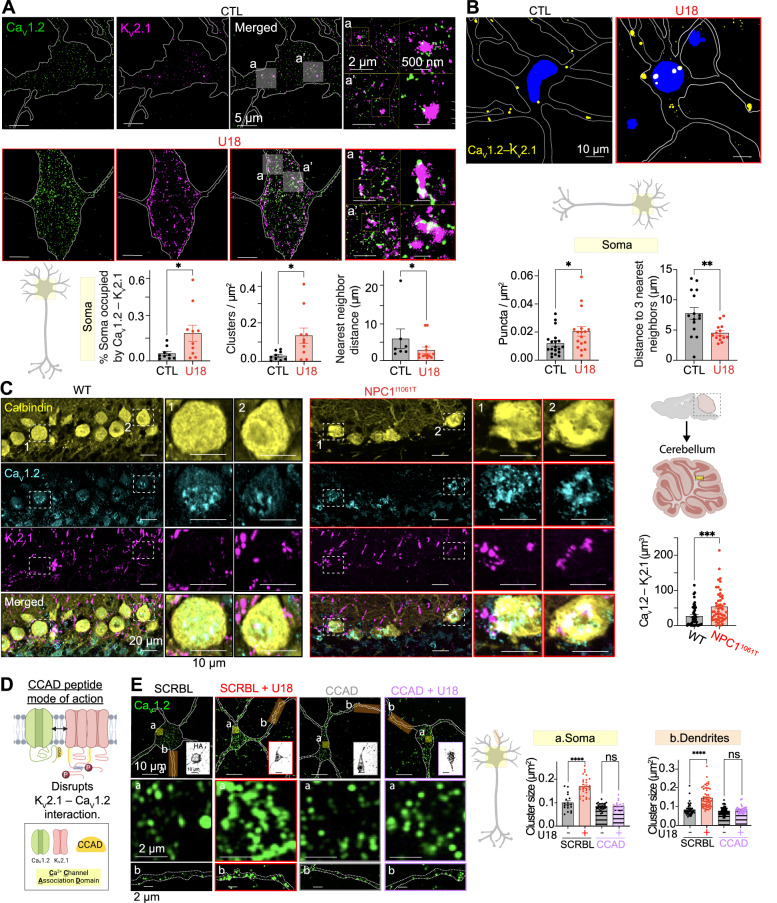
NPC1 inhibition and NPC1 disease mutations increase spatial proximity between K_V_2.1–Ca_V_1.2. **A** *Top*, representative super-resolution TIRF images of CTL (black) and U18-treated (red) neurons co-immunolabeled for Ca_V_1.2 and K_V_2.1. *Bottom*, quantification of % of soma occupied by K_V_2.1–Ca_V_1.2, K_V_2.1–Ca_V_1.2 cluster density and K_V_2.1–Ca_V_1.2 nearest cluster distance of CTL (black) and U18-treated (red) neurons in the soma region. *N* = 9 (CTL) and *n* = 10 (U18) neurons were analyzed across 2 independent isolations. **B** *Top*, representative Ca_V_1.2 and K_V_2.1 PLA images of CTL (black) and U18-treated (red) neurons. *Bottom*, quantification of PLA puncta density and nearest puncta distance of CTL (black) and U18-treated (red) neurons. *N* = 19 (CTL) and *n* = 17 (U18) neurons were analyzed across 2 independent isolations. **C** *Left*, representative maximum intensity projections from WT (black) and NPC1^I1061T^ (red) cerebellar sagittal sections co-immunolabeled for Calbindin, Ca_V_1.2 and K_V_2.1. *Right*, quantification of K_V_2.1–Ca_V_1.2 colocalization volume in WT (black) and NPC1^I1061T^ (red) neurons in the soma region. *n* = 47–48 (WT and NPC1^I1061T^) neurons were analyzed across 3 animals. **D** Diagram detailing HA-TAT-Ca^2+^ Channel Association Domain (CCAD) mode of action. **E** *Left*, representative super-resolution Airyscan images taken at a focal plane near the PM of CTL (black) and U18 (red) neurons incubated with the CCAD or HA-TAT-Scr scrambled peptide (SCRBL), and co-immunolabeled for Ca_V_1.2 and HA. *Right*, quantification of PM Ca_V_1.2 cluster size of CTL (black) and U18-treated (red) neurons co-incubated with CCAD or SCRBL peptide in the soma (left, yellow) and dendrite (right, orange) regions. *N* = 18 (SCRBL), *n* = 30 (SCRBL + U18), *n* = 25 (CCAD) and *n* = 19 (CCAD + U18) neurons and *n* = 46 (SCRBL), *n* = 55 (SCRBL + U18), *n* = 57 (CCAD) and *n* = 34 (CCAD + U18) dendrites were analyzed across 2 independent isolations. All error bars represent SEM. Statistical significance was calculated using Mann–Whitney (two-tail) and Unpaired *t* tests (two-tail) in (**A**)–(**C**) and two-way ANOVA in (**E**); ns: not significant; *P < 0.05; **P < 0.01; ***P < 0.001; ****P < 0.0001. CTL is control, SCRBL is scramble, U18 is U18666A, and CCAD is calcium channel association domain.

Next, to test if animal models of NPC1 disease also have altered neuronal K_V_2.1 distribution, we fixed, sectioned, and immunolabeled brain sections from WT and NPC1^I1061T^ (most prevalent disease mutation) mice for K_V_2.1. Similar to treatment with U18, mice harboring the NPC1^I1061T^ mutation displayed increased K_V_2.1 clustering in cortical (Fig. 3C) and hippocampal (Fig. S3B) pyramidal neurons, and cerebellar Purkinje neurons (Fig. 4D). Therefore, like Ca_V_1.2, K_V_2.1 clusters are larger and more abundant in NPC disease.

### NPC1 loss of function increases K_V_2.1–Ca_V_1.2 colocalization in vitro and in vivo

Loss of NPC1 function increases Ca_V_1.2 (Fig. 2) and K_V_2.1 (Fig. 3) clustering in somatic regions of neurons. Given that these two channels physically interact^31^, we used two complementary approaches to test if loss of NPC1 function alters the proximity of K_V_2.1–Ca_V_1.2 channel complexes. First, using super-res_TIRF_ imaging we quantified the relative distribution between Ca_V_1.2 and K_V_2.1 channel complexes. Figure 4A shows that around 0.05% of neuronal somatic pixels were positive for both Ca_V_1.2 and K_V_2.1 in control conditions at this enhanced resolution. Inhibition of NPC1 led to a 3-fold increase in the number of somatic pixels positive for both Ca_V_1.2 and K_V_2.1, with increases in the average size of Ca_V_1.2–K_V_2.1 clusters (Fig. 4A). Furthermore, NPC1 loss-of-function also reduced the average distance between adjacent Ca_V_1.2–K_V_2.1 hetero-clusters (Fig. 4A). Identical results were observed in blinded experimental datasets (Fig. S4A, B) and NPC1^−/−^ cells (Fig. S4C). To further elucidate if the physical proximity between Ca_V_1.2 and K_V_2.1 is altered in NPC1 disease conditions, we performed Proximity Ligation Assays (PLA). The PLA technique allows the detection and quantification of those Ca_V_1.2 and K_V_2.1 channels that are within 40 nm of one another^63^. Like super-res_TIRF_ imaging, PLA experiments revealed an increase in the number of Ca_V_1.2–K_V_2.1 channel clusters within 40 nm of one another, with each hetero-cluster being closer to each other in the neuronal soma following loss of NPC1 function (Fig. 4B).

To corroborate that increased Ca_V_1.2–K_V_2.1 complexes occur in intact brain regions, we performed multiplexed immunolabeling on cerebellar tissue of WT and NPC1^I1061T^ mutant mice (Fig. 4C). The cerebellum was chosen because Purkinje cells represent another highly vulnerable neuronal population in NPC1 disease^64,65^. Like isolated cortical neurons, Ca_V_1.2 and K_V_2.1 channels showed increased spatial proximity in mouse NPC1^I1061T^ Purkinje neurons (Fig. 4C; movie S1). Thus, both NPC1 inhibition and disease mutations increase Ca_V_1.2–K_V_2.1 complexes.

K_V_2.1 channels associate with Ca_V_1.2 channels via a Ca^2+^ Channel Association Domain (CCAD) located in the proximal cytoplasmic C-terminus of K_V_2.1^2^. To test if CCAD-dependent interactions between K_V_2.1–Ca_V_1.2 drive increases in Ca_V_1.2 channel clusters following loss of NPC1 function we took advantage of a cell permeant synthetic peptide “HA-TAT-C1aB” (CCAD)^66^ which selectively uncouples K_V_2.1–Ca_V_1.2 channel complexes^2^. Using this synthetic tool, and a corresponding peptide with a scrambled CCAD sequence “TAT-HA-C1aB-Scr” (SCRBL), we compared Ca_V_1.2 distribution in isolated mouse (Fig. 4D, E and Fig. S4B) and rat (Fig. S5A) neurons under control and U18 conditions, as well as NPC1^−/−^ cells (Fig. S4C). Analysis revealed that in all models increases in Ca_V_1.2 density and cluster size were eliminated in the presence of CCAD, but not the Scr control peptide, providing evidence that U18 or NPC1^−/−^ –associated elevations in the size of Ca_V_1.2 clusters are dependent on interactions with K_V_2.1 channels.

### CDK5-dependent phosphorylation of K_V_2.1 drives NPC1-dependent increases in Ca_V_1.2 clustering in isolated neurons

**Fig. 5:**
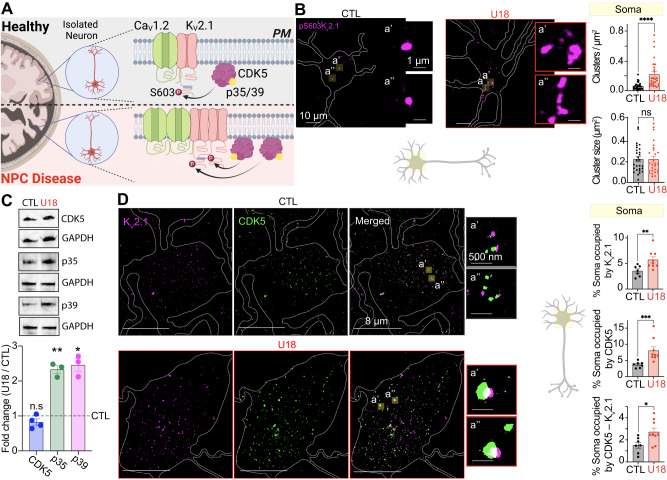
Inhibition of NPC1 increases p35/p39 protein expression and CDK5 proximity to KV2.1. **A** Schematic diagram of the hypothesis: CDK5 drives enhanced K_V_2.1 phosphorylation in NPC disease. **B** *Left*, representative super-resolution Airyscan images taken at a focal plane near the PM of CTL (black) and U18-treated (red) neurons fixed and immunolabeled for K_V_2.1p(S603). *Right*, quantification of PM K_V_2.1p(S603) cluster density and cluster size of CTL (black) and U18-treated (red) neurons in the soma region. *N* = 28 (CTL) and *n* = 27 (U18) neurons were analyzed across two independent isolations. **C** *Top*, representative blots of CDK5, p35, p39 with their respective GAPDH. *Bottom*, quantification of CDK5, p35 and p39 fold change (U18/CTL) normalized to GAPDH. *N* = 4 (CDK5), *n* = 3 (p35), and *n* = 3 (p39) across independent isolations. **D** *Left*, representative super-resolution TIRF images of CTL (black) and U18-treated (red) neurons co-immunolabeled for K_V_2.1 and CDK5. *Right*, quantification of % of the soma occupied by K_V_2.1, CDK5, and CDK5–K_V_2.1. *N* = 7 (CTL) and *n* = 9 (U18) neurons were analyzed across 2 independent isolations. All error bars represent SEM. Statistical significance was calculated using Mann–Whitney (two-tail) and Unpaired *t* test (two-tail) in (**B**) and (**D**), and one Sample *t* test in (**C**); ns: not significant; *P < 0.05; **P < 0.01; ***P < 0.001; ****P < 0.0001. CTL is control, U18 is U18666A, CDK5 is Cyclin dependent kinase 5.

Clustering of PM K_V_2.1 channels is regulated by phosphorylation^32,67^ with protein kinases like Cyclin Dependent Kinase 5 (CDK5) promoting K_V_2.1 clustering^67^ (Fig. 5A), and protein phosphatases like calcineurin de-clustering of K_V_2.1^32^. To test if loss of NPC1 function increases phosphorylation of K_V_2.1 channels we fixed and immunolabeled neurons with a phospho-specific antibody against the pS603 site of K_v_2.1 which has been shown to be sensitive to CDK5 phosphorylation^67^. Subsequent super-res_TIRF_ analysis revealed the density of somatic K_V_2.1 pS603 clusters to be significantly increased in U18 treated neurons relative to control (Fig. 5B). To test if CDK5 underlies increased K_V_2.1 phosphorylation in NPC disease we first measured CDK5 protein expression and found it was similar between control and U18 treated neurons (Fig. 5C). Next, we asked if CDK5 activating proteins p35/p39 have altered expression following loss of NPC1 function. Consistent with a report of increased CDK5 enzymatic activity in NPC disease models^68^, expression of both p35 and p39 proteins was significantly elevated following inhibition of NPC1 (Fig. 5C). Interactions between CDK5 and p35 promote targeting and anchoring of the enzyme to the plasma membrane^69,70^; therefore, we performed dual-label super-res imaging for K_V_2.1 and CDK5 and found the fractional amount of CDK5 overlapping with K_V_2.1 increased when NPC1 was inhibited (Fig. 5D). Collectively, these experiments support a model whereby NPC1 loss-of-function increases p35/p39 expression which preferentially targets CDK5 to the plasma membrane. Here it is in closer proximity to K_V_2.1 to increase phosphorylation-dependent clustering of K_V_2.1 thereby promoting increased K_V_2.1–Ca_V_1.2 interactions.

### Targeting CDK5 rescues clustering phenotypes in NPC disease in vitro

**Fig. 6:**
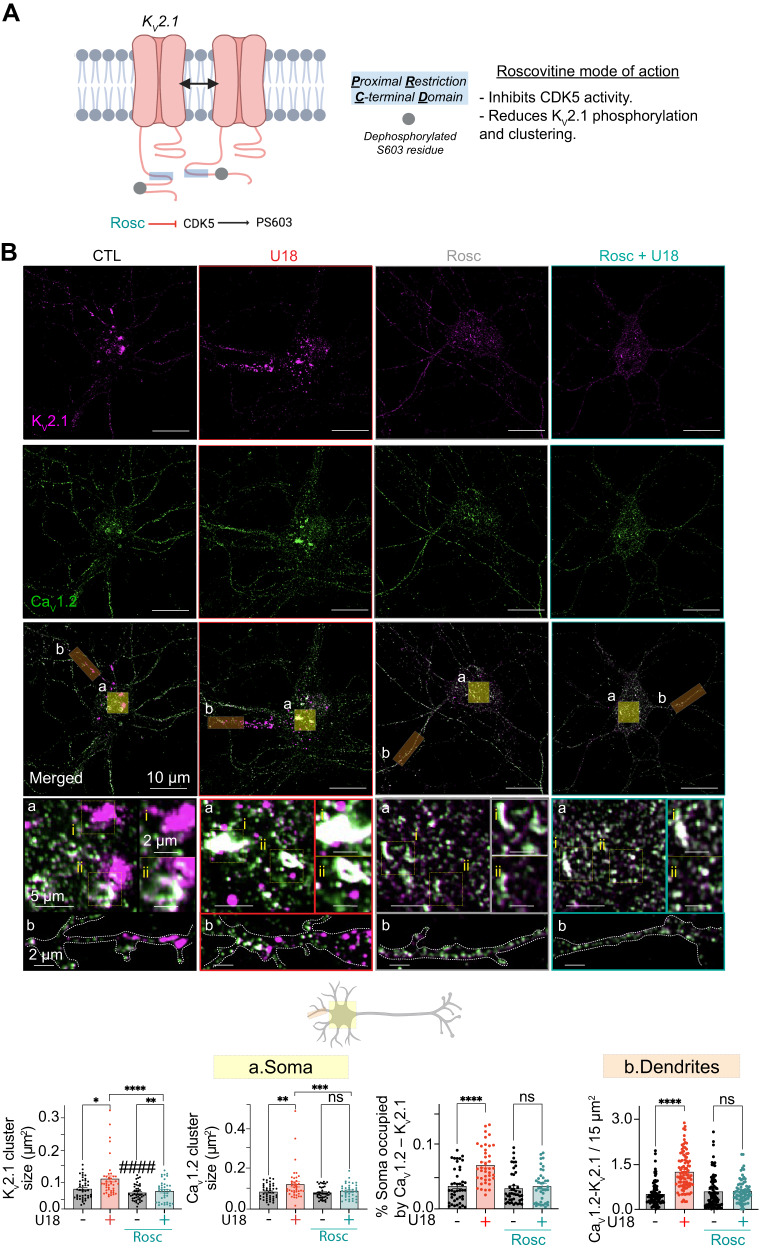
NPC1-dependent increases in Ca_V_1.2 are abrogated by inhibiting CDK5-dependent phosphorylation of K_V_2.1. **A** Schematic diagram detailing roscovitine (Rosc) mode of action. **B** *Top*, representative super-resolution Airyscan images taken at a focal plane near the PM of CTL (black) or U18-treated (red) neurons co-incubated with roscovitine (Rosc) (cyan) and co-immunolabeled for Ca_V_1.2 and K_V_2.1. *Bottom*, quantification of PM KV2.1 and Ca_V_1.2 clustering size, and % of the soma occupied by Ca_V_1.2–K_V_2.1 (left, yellow) and Ca_V_1.2–K_V_2.1 area in the dendrite region (right, orange) of CTL (black), U18 (red) and Rosc (cyan) neurons. *N* = 43 (CTL), *n* = 41 (U18), *n* = 43 (Rosc) and *n* = 38 (Rosc+U18) neurons and *n* = 89 (CTL), *n* = 94 (U18), *n* = 82 (Rosc) and *n* = 71 (Rosc+U18) dendrites were analyzed across 5 independent isolations. All error bars represent SEM. Statistical significance was calculated using a two-way ANOVA test. ns: not significant; *P < 0.05; **P < 0.01; ***P < 0.001; ****P < 0.0001.^#^*P* < 0.05; ^##^*P* < 0.01; ^####^*P* < 0.0001. ^#^ indicates comparison with the CTL condition. CTL is control, U18 is U18666A, ROSC is Roscovitine.

To probe more directly that CDK5 activity drives phosphorylation of K_V_2.1 to enhance K_V_2.1–Ca_V_1.2 complexes in models of NPC1 we conducted experiments using the small molecule CDK5 inhibitor, roscovitine (Fig. 6A). Cortical neurons were incubated with U18 and Roscovitine, before being fixed and immunolabeled for Ca_V_1.2 and K_V_2.1. In agreement with super-res analysis of K_V_2.1 pS603 (Fig. 5B), U18 treatment significantly increased the area occupied by both Ca_V_1.2 and K_V_2.1 in somatic and proximal dendrites (Fig. 6B). In contrast, inhibiting CDK5 not only prevented the U18-dependent increases in both K_V_2.1 and Ca_V_1.2 clustering (Fig. 6B) but also abrogated U18-mediated elevations in Ca_V_1.2–K_V_2.1 hetero-cluster size and density (Fig. 6B). Interestingly, roscovitine did not alter steady-state K_V_2.1–Ca_V_1.2 hetero-cluster sizes in control neurons, suggesting a CDK5-independent mechanism for recruitment and binding of K_V_2.1 to Ca_V_1.2. This is perhaps not surprising given that several protein kinases can phosphorylate K_V_2.1 at different positions and supports the concept that multiple protein kinases may tune K_V_2.1–Ca_V_1.2 interactions. As a final test, we began to probe molecular elements upstream of CDK5–p35/p39. To this end, we focused on mTORC1 as it has been shown to be hyperactive in NPC disease^15^ and is proposed to inhibit AMPK^71^ leading to increased CDK5 activity^72^. To test for an upstream role for mTORC1 we treated neurons with a combination of U18 (to inhibit NPC1) and/or Torin-1 (to inhibit mTORC1) before fixing and labeling for Ca_V_1.2 and K_V_2.1. Subsequent super-res imaging revealed that U18-dependent increases Ca_V_1.2 and K_V_2.1 clusters were sensitive to mTORC1 inhibition with K_V_2.1–Ca_V_1.2 nanocomplexes reduced back to control levels (Figure S5B). These data suggest that mTORC1 may be an upstream element controlling K_V_2.1–Ca_V_1.2 complexes in NPC disease. More experiments are required to determine the precise molecular pathway linking mTORC1 to alterations in K_V_2.1–Ca_V_1.2 complexes.

### K_V_2.1-associated Ca_V_1.2 influences the formation of ER–PM junctions

**Fig. 7:**
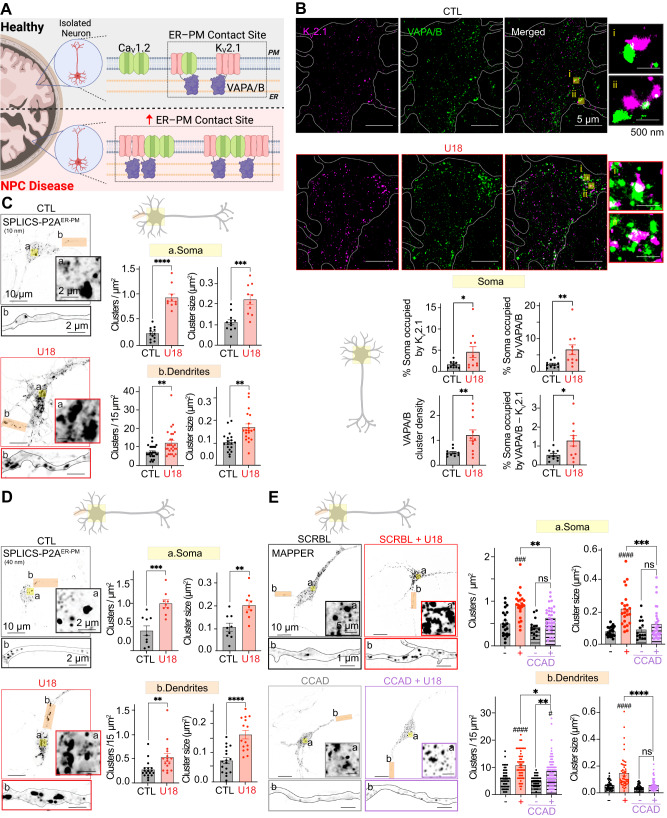
NPC1 regulates the size and density of K_V_2.1–VAPA/B and ER–PM MCSs. **A** Schematic diagram of the hypothesis: loss of NPC1 function leads to increased ER-PM junctions. **B** *Top*, representative super-resolution TIRF images of CTL (black) and U18-treated (red) neurons co-immunolabeled for K_V_2.1 and VAPA/B. *Bottom*, quantification of % of the soma occupied by K_V_2.1, VAPA/B, and K_V_2.1–VAPA/B as well as VAPA/B cluster density. *N* = 10 (CTL) and *n* = 11 (U18) neurons were analyzed across 2 independent isolations. **C** *Left*, representative images taken at a focal plane near the PM of CTL (black) and U18 (red) neurons transfected with SPLICS_S_-P2A^ER-PM^ probe (10 nm). *Right*, quantification of SPLICS_L_-P2A^ER-PM^ cluster density and size of CTL (black) and U18 (red) neurons in the soma (top, yellow) and dendrite (bottom, orange) regions. *N* = 9 (CTL) and *n* = 9 (U18) neurons and *n* = 17 (CTL) and *n* = 15 (U18) dendrites were analyzed across one isolation. **D** Same as (**C**), only transfected with SPLICS_L_-P2A^ER-PM^ probe (40 nm). *N* = 12 (CTL) and *n* = 10 (U18) neurons and *n* = 19 (CTL) and *n* = 20 (U18) dendrites were analyzed across one isolation. **E** *Left*, representative images taken at a focal plane near the PM of MAPPER transfected CTL (black), U18 (red) and CCAD + U18 (purple) neurons. Bottom, quantification of MAPPER puncta density and puncta size of CTL (black), U18 (red) and CCAD + U18 (purple) neurons in the soma (top, yellow) and dendrite (bottom, orange) regions. *N* = 22 (CTL), *n* = 23 (U18), *n* = 15 (CCAD) and *n* = 30 (CCAD + U18) neurons and *n* = 47 (CTL), *n* = 50 (U18), *n* = 34 (CCAD) and *n* = 72 (CCAD + U18) dendrites were analyzed across 3 independent isolations. All error bars represent SEM. Statistical significance was calculated using Mann–Whitney (two-tail) and unpaired *t* test (two-tail) in (**B**)–(**D**) and two-way ANOVA in (**E**). ns: not significant; *P < 0.05; **P < 0.01; ***P < 0.001; ****P < 0.0001. ^#^*P* < 0.05; ^##^*P* < 0.01; ^####^*P* < 0.0001. ^#^ indicates comparison with the CTL condition. CTL is control, SCRBL is scramble, U18 is U18666A, and CCAD is calcium channel association domain.

Phosphorylation of K_V_2.1 within its Proximal Restriction and Clustering (PRC) domain generates a noncanonical FFAT (two phenylalanines (FF) in an Acidic Tract) motif that facilitates interactions with ER VAP proteins leading to the formation of ER–PM MCSs^34,35^. Given the increase in K_V_2.1 clusters following loss of NPC1 function, we asked if ER–PM MCSs are also altered (Fig. 7A). To begin we performed a volumetric analysis using a live-neuron ER dye to test if basic ER morphology is altered following NPC1 inhibition and determined that ER filament length and density, as well as ER branching density were the same in control and U18-treated neurons (Fig. S6). Therefore, NPC1 inhibition does not appear to result in gross changes to ER morphology. Next, we performed dual-label immunofluorescence against K_V_2.1 and VAPA/B and determined using super-res_TIRF_ imaging that VAPA/B cluster density, size, and overlap with K_V_2.1 were all increased when NPC1 was inhibited (Fig. 7B). These data are consistent with increased ER–PM contact sites in NPC disease models. To test this hypothesis, we took advantage of two fluorescent tools: (i) SPLICS_S/L_-P2A^ER-PM^ ^73^ and (ii) MAPPER^74^, which enable visualization and quantification of ER–PM MCSs in living neurons. The SPLICS_S/L_-P2A^ER-PM^ tool is based on the split-YFP protein fused to either an ER or PM target so it only emits fluorescence upon self-assembly at the ER–PM interface. A short or long spacer placed at the split-YFP ER targeted protein allows for the study of narrow (8–10 nm) or wide (40–50 nm) ER–PM MCSs^73^. Transfection of this ER-PM sensor into cortical neurons revealed a punctate distribution within either 40 nm or 10 nm of the TIRF footprint that represents the cortical ER in close apposition to the PM (Fig. 7C, D). Comparison of control and U18-treated neurons demonstrated that loss of NPC1 function resulted in an increase in the amount of ER in close proximity to the PM (Fig. 7C, D). As an alternative approach to quantify changes in ER–PM MCSs we used the MAPPER construct. The MAPPER tool is a GFP-tagged single peptide that contains the transmembrane domain of STIM1 (targeted to the ER) and a polybasic motif of the small G-protein Rit (PM binding)^74^. The polybasic motif interacts with negatively charged lipids at the cytoplasmic leaflet of the PM and enables visualization of ER–PM contacts between 10 and 25 nm. Like SPLICS_S/L_-P2A^ER-PM^, expression of MAPPER revealed a punctate distribution when visualized using super-res AiryScan microscopy at a focal plane close to the PM (Fig. 7E). Quantification of MAPPER images determined that MAPPER puncta density and size were both increased in U18-treated neurons at the soma and dendrites (Fig. 7E). Interestingly, when K_V_2.1–Ca_V_1.2 interactions were disrupted with the CCAD peptide, U18-dependent increases in ER–PM MCSs were partially reversed with decreases in the number and size of MAPPER puncta (Fig. 7E). These data suggest that K_V_2.1-associated Ca_V_1.2 plays an important role in generating ER–PM MCSs in NPC1 neurons.

### NPC1 inhibition remodels Ca^2+^ handling proteins at ER–PM MCSs to potentiate Ca^2+^ signaling in vitro

**Fig. 8:**
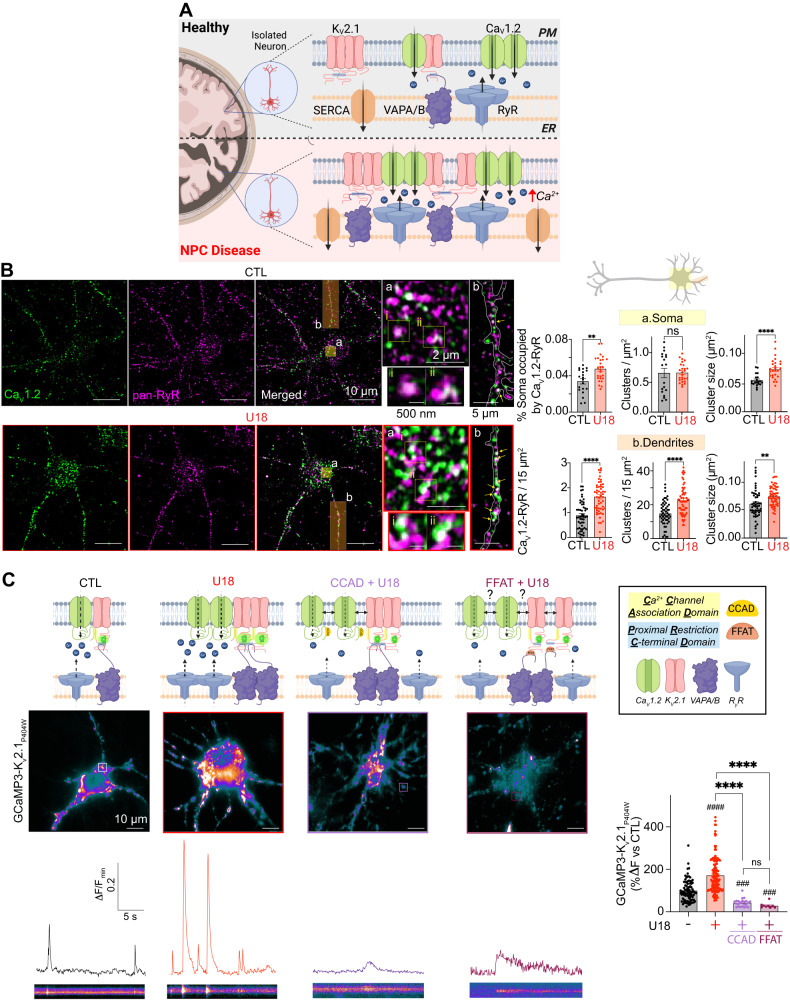
Loss of NPC1 function spatially and functionally remodels RyR–Ca_v_1.2–K_V_2.1 signaling nanodomains. **A** Schematic diagram of the hypothesis: NPC1 deficient neurons have ER-PM domains enriched in RyR—Ca_V_1.2–K_v_2.1—SERCA and increased spontaneous Ca^2+^ activity at K_V_2.1-asociated ER**–**PM MCSs. **B** *Left*, representative super-resolution Airyscan images taken at a focal plane near the PM of CTL (black) and U18-teated (red) neurons co-immunolabeled for Ca_V_1.2 and RyR. *Right*, quantification of % soma occupied by Ca_V_1.2–RyR, Ca_V_1.2–RyR cluster density and Ca_V_1.2–RyR cluster size of CTL (black) and U18-treated (red) neurons in the soma (top, yellow) and dendrite (bottom, orange) region. *N* = 20–21 (CTL) and *n* = 26 (U18) neurons and *n* = 52–53 (CTL) and *n* = 54–55 (U18) dendrites were analyzed across 2 indepdendent isolations. **C** *Top*, schematic diagram of hypothesis: NPC1 deficient neurons (U18, red) have increased Ca^2+^ activity and K_V_2.1-associated Ca_V_1.2, while disrupting K_v_2.1–Ca_v_1.2 or K_V_2.1–VAPA/B interactions (CCAD, purple or FFAT, red-brown) abrogates such effects. *Middle left*, representative super-resolution TIRF images of neurons transfected with GCamP3-Kv2.1_P4O4W_. *Bottom left*, intensity time series and kymographs of spontaneous activity taken from the square region of interest. *Middle right*, quantification of GCamP3-Kv2.1_P4O4W_ peak amplitude of CTL (black), U18 (red), CCAD + U18 (purple) and FFAT + U18 (red-brown) neurons. *N* = 85 (CTL), *n* = 141 (U18), *n* = 23 (CCAD + U18) and *n* = 10 (FFAT + U18) GCamP3-Kv2.1_P4O4W_ peaks were analyzed across 3 independent isolations. All error bars represent SEM. Statistical significance was calculated using the following tests: Unpaired (two-tail) and Mann–Whitney *t* tests (two-tail) in (**B**) and Kruskal–Wallis test in (**C**). ns: not significant; *P < 0.05; **P < 0.01; ***P < 0.001; ****P < 0.0001. ^#^*P* < 0.05; ^##^*P* < 0.01; ^####^*P* < 0.0001. ^#^ indicates comparison with the CTL condition. CTL is control, SCRBL is scramble, U18 is U18666A, CCAD is calcium channel association domain, and FFAT is two phenylalanines (FF) in an acidic tract.

At somatic ER–PM MCSs, in addition to Ca_V_1.2, K_V_2.1 is in close spatial proximity with several other Ca^2+^ handling proteins, including Ryanodine Receptors (RyR) and Sacro/Endoplasmic Reticulum Ca^2+^-ATPase (SERCA)^31,75^. Considering the increases in K_V_2.1–Ca_V_1.2 complexes (Fig. 4) and ER–PM MCSs (Fig. 7) observed following the loss of NPC1 function we wanted to determine if RyR and SERCA are enriched at these ER–PM contacts (Fig. 8A). To test if SERCA distribution is altered at K_V_2.1-forming nanodomains we performed super-res_TIRF_ on neurons fixed and co-immunolabeled for K_V_2.1 and SERCA. In control and U18-treated neurons SERCA was observed in the TIRF footprint, indicating close spatial proximity to the PM (Fig. S7A). Furthermore, U18 treatment significantly enhanced SERCA cluster area and overlap with K_V_2.1 (Figure S7A). These data align with previously published results that SERCA expression is significantly increased in NPC disease models^23^. To assess RyR distribution relative to K_V_2.1–Ca_V_1.2 domains, we co-immunolabeled for Ca_V_1.2 and RyR with or without U18 treatment to inhibit NPC1. Using super-res AiryScan confocal microscopy we quantified RyR and Ca_V_1.2 distribution at a focal plane close to the PM and found that in addition to expected increases in Ca_V_1.2 cluster size, their proximity to one another increased when NPC1 function is lost (Fig. 8B). On average, there was a 40 % increase in overlapping Ca_V_1.2–RyR pixels in the soma and a doubling of overlapping pixels in the dendrites of NPC loss of function neurons relative to control. These findings reveal that both RyR and SERCA are enriched at K_V_2.1 associated ER–PM MCSs following the loss of NPC1 function.

Considering the importance of K_V_2.1 for tuning ER–PM Ca^2+^ nanodomains^2,31^ and the significant reorganization of K_V_2.1–Ca_V_1.2–RyR–SERCA that occurs following loss of NPC1 function, we next wanted to understand if this molecular remodeling results in augmented Ca^2+^ signaling at these K_V_2.1 associated ER–PM contacts. To monitor and quantify Ca^2+^ signaling at K_V_2.1 ER–PM MCSs we took advantage of a recently described GCaMP Ca^2+^ sensor, GCaMP3-K_v_2.1_P4O4W_ which is GCaMP3 appended to the N-terminus of nonconducting K_V_2.1^31^. Transfection of GCaMP3-K_v_2.1_P4O4W_ into cortical neurons resulted in its distinct localization to ER–PM MCSs (Fig. 8C). Using TIRF microscopy to visualize GCaMP3-K_v_2.1_P4O4W_ in close apposition to the PM we quantified spontaneous changes in GCaMP3 intensity from neurons expressing low amounts of the Ca^2+^ biosensor. Analysis of GCaMP3s signals determined that in NPC loss of function neurons, the amplitude of spontaneous Ca^2+^ signals at K_V_2.1-forming ER–PM MCSs effectively doubled compared to control (Fig. 8C). To test the role of Ca_V_1.2 channels in facilitating this increase we uncoupled K_V_2–Ca_V_1 interactions at ER–PM MCS (co-incubation with the CCAD peptide) which resulted in neurons being refractory to U18 and having significantly decreased responses relative to control (Fig. 8C; Movie S2). Similar results were observed when neurons were treated with a cell-penetrating FFAT peptide to uncouple K_V_2.1 from VAPA/B. Finally, to test if CCAD decreased Ca^2+^ elevations near K_V_2.1 due to selective uncoupling of K_V_2.1–Ca_V_1.2 or due to hyperpolarization in the membrane potential we performed experiments using a genetically encoded voltage indicator (ArchLight-Q239^76^). High-speed imaging of ArchLight-Q239 revealed that U18-treated neurons had similar fluctuations in intensity when compared to U18 and CCAD (Fig. S7B), suggesting that CCAD-dependent decreases in Ca^2+^ (Fig. 8D) occur through selective uncoupling of K_V_2.1–Ca_V_1.2 rather than alterations in neuronal excitability.

Taken together, these results demonstrate a role for NPC1 in the downstream organization of spatial and functional coupling between PM K_V_2.1–Ca_V_1.2 and the ER to regulate ER–PM Ca^2+^ nanodomains.

### Remodeling of ER–PM and ER–Mito MCSs results in neurotoxic increases in mitochondrial Ca^2+^ and neurodegeneration

**Fig. 9:**
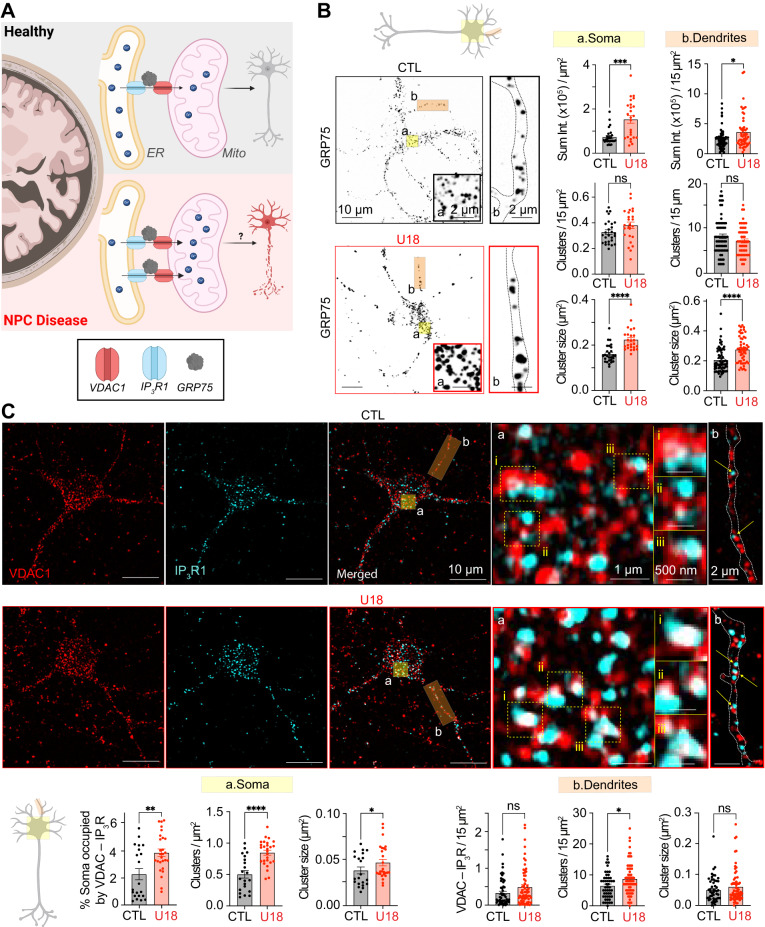
NPC1 dysfunction increases GRP75 and IP_3_R1–VDAC1 clustering. **A** Schematic diagram of the hypothesis: Enhanced IP_3_R–GRP75–VDAC signaling axis in NPC neurons leads to an aberrantly increased mitochondrial Ca^2+^ and neurotoxicity. **B** Left, representative super-resolution Airyscan images of CTL (black) and U18-treated neurons immunolabeled for GRP75. *Right*, quantification of GRP75 total intensity, cluster density and cluster size of CTL (black) and U18 (red) neurons in the soma (left, yellow) and dendrite (right, orange) regions. *N* = 27 (CTL) and *n* = 25 (U18) neurons and *n* = 65 (CTL) and *n* = 54–55 (U18) dendrites were analyzed across 2 independent isolations. **C** *Top*, representative images of CTL (black) and U18 (red) neurons fixed and immunolabeled for IP_3_R and VDAC1. *Bottom*: quantification of % soma occupied by IP_3_R–VDAC1, IP_3_R–VDAC1 cluster density and IP_3_R–VDAC1 cluster size of CTL (black) and U18-treated (red) neurons in the soma (left, yellow) and dendrite (right, orange) regions. *N* = 21 (CTL) and *n* = 27 (U18) neurons and *n* = 48 (CTL) and *n* = 65 (U18) dendrites across 2 independent isolations. All error bars represent SEM. Statistical significance was calculated using Mann–Whitney (two-tail) and Unpaired *t* tests (two-tail) in (**B**) and (**C**). ns: not significant; *P < 0.05; **P < 0.01; ***P < 0.001; ****P < 0.0001. CTL is control and U18 is U18666A.

Our data suggest there are significant alterations to global and local (ER–PM MCSs) Ca^2+^ nanodomains in NPC neurons. Considering the importance of appropriately regulated Ca^2+^ levels for neuronal signaling, we next wanted to determine if these excessive Ca^2+^ events had toxic consequences for neuronal viability. In neurons, the ER and mitochondria act as major buffers in response to high cytosolic Ca^2+^ levels. Functional coupling between the IP_3_R and the Voltage-Dependent Anion channel 1 (VDAC1) at ER–Mito MCSs ensures a steep local Ca^2+^ gradient for Ca^2+^ to move into mitochondria to maintain energy production and cell health^77,78^. However, deviant mitochondrial Ca^2+^ (Ca^2+^_Mito_) levels can lead to altered bioenergetics, ATP production, and Reactive Oxygen Species (ROS) production, triggering necrosis or apoptosis^79–81^. Therefore, we tested the hypothesis that NPC1-dependent elevations in cytoplasmic Ca^2+^ increases Ca^2+^_Mito_ and leads to neurotoxicity (Fig. 9A).

We began by characterizing the gross structural organization of the ER and mitochondria in control and U18-treated cells loaded with mitochondria- and ER-permeable dyes. Super-res AiryScan microscopy was performed to measure the overlapping signal near the PM. These analyses demonstrated that the proportion of ER overlapping with mitochondria was very similar in both conditions (Fig. S8A), suggesting that at this resolution there are no obvious rearrangements of these organelle membranes. Next, we asked more specifically if ER–Mito Ca^2+^ domains were affected by NPC1 function. At ER–Mito MCSs the IP_3_R and VDAC1 are coupled through interactions with the chaperone protein GRP75^82^. To determine if the IP_3_R–GRP75–VDAC1 signaling axis, which has been implicated in the progression of other neurodegenerative diseases like Alzheimer’s, Parkinson’s, and Amyotrophic Lateral Sclerosis, undergoes reorganization in NPC disease we took a two-pronged approach leveraging NPC1^−/−^ cells and neurons treated with U18. First, using control and NPC1^-/-^ cells, or vehicle and U18-treated neurons, we immunolabeled for the GRP75 protein and acquired super-resolution AiryScan confocal images. Fig. 9B shows that the total fluorescence intensity, cluster density and cluster size was augmented in U18-treated neurons compared to control. Similarly, NPC1^−/−^ cells also showed higher GPR75 intensity levels and larger clusters (Fig. S8B), indicating enhanced GPR75 at ER–Mito MCSs.

To understand if increased GRP75 cluster size and intensity also reflects remodeling of IP_3_R and VDAC1 proteins at ER–Mito MCSs we quantified the overlap between these proteins using two models of NPC disease. First, using neurons, we fixed and co-immunolabeled for VDAC1 and IP_3_R1 before imaging using super-resolution Airyscan confocal microscopy. As shown in Fig. 9C, analyses of resultant 2-color images determined that the proportion, density, and size of VDAC1–IP_3_R overlapping clusters were all significantly increased in U18 conditions. Similar experiments performed using NPC1^−/−^ cells (Fig. S8C), HEK cells that have endogenous IP_3_R1 tagged with eGFP (Fig. S8D^83^), and blinded neuronal experiments (Fig. S8E) revealed that NPC1 knockout or inhibition increased the amount of overlapping IP_3_R–VDAC1. Taken together, our data indicated that the IP_3_R–GPR75–VDAC1 signaling axis is upregulated following NPC1 loss-of-function, suggesting Ca^2+^ flux from ER to mitochondria may be altered in NPC disease.

**Fig. 10:**
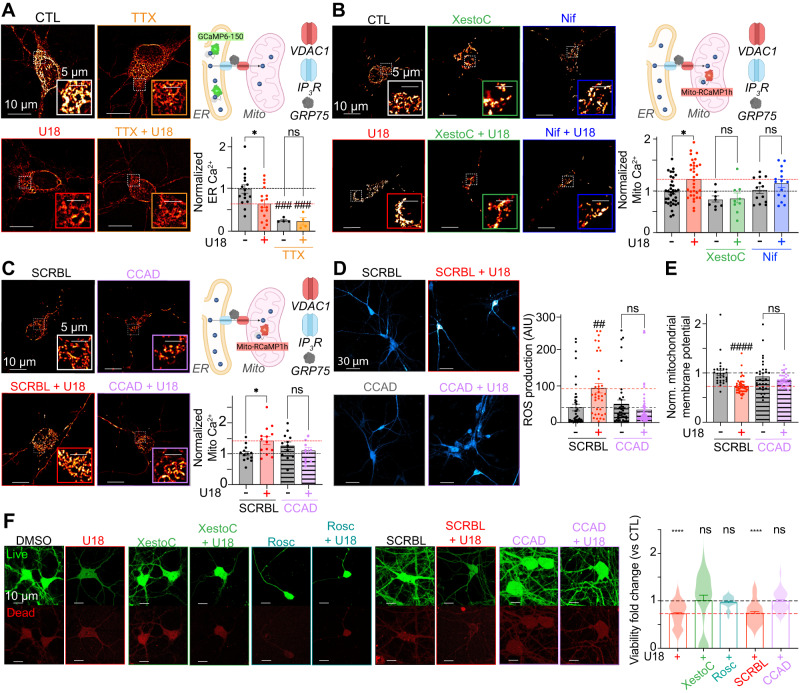
Targeting K_V_2.1–Ca_V_1.2 interactions reduces Mito Ca^2+^ and rescues neurotoxicity in NPC disease. **A** *Left*, representative images of neurons transfected with ER-GCaMP6-150. *Top right*, ER-GCaMP6-150 localization. *Bottom right*, ER Ca^2+^ quantifications. *N* = 16 (CTL), *n* = 17 (U18), *n* = 4 (TTX) and *n* = 4 (TTX + U18) neurons were analyzed across 2 independent isolations. **B** *Left*, representative images of CTL (black), U18 (red), nifedipine (Nif) (blue) and Xestospongin C (XestoC) (green) neurons transfected with Mito-RCamP1h. *Right top*, Mito-RCamP1h localization. *Right bottom*, normalized Mito Ca^2+^. *N* = 35 (CTL), *n* = 33 (U18), *n* = 13 (Nif), *n* = 15 (Nif+U18), *n* = 8 (XestoC) and *n* = 9 (XestoC+U18) neurons were analyzed across 3 independent isolations. **C** *Left*, neurons expressing Mito-RCamP1h. *Right top*, Mito-RCamP1h localization. *Right bottom*, normalized Mito Ca^2+^. *N* = 13 (SCRBL), *n* = 14 (SCRBL + U18), *n* = 12 (CCAD) and *n* = 12 (CCAD + U18) neurons were analyzed across 2 independent isolations. **D** *Left*, representative images of neurons incubated with H2DCFDA. *Right*, quantification of ROS. *N* = 43 (SCRBL), *n* = 36 (SCRBL + U18), *n* = 39 (CCAD) and *n* = 64 (CCAD + U18) neurons were analyzed across 2 independent isolations. **E** Quantification of neuron mitochondrial membrane polarization. *N* = 28 (SCRBL; black), *n* = 48 (SCRBL + U18; red), *n* = 30 (CCAD; black) and *n* = 39 (CCAD + U18; purple) neurons were analyzed across 2 independent isolations. **F** Left, representative images of Live and dead channels from neurons in each condition. Quantification of neuronal viability. *N* = 394 (DMSO), 174 (U18), *n* = 94 (XestoC), *n* = 28 (XestoC + U18), *n* = 124 (SCRBL), *n* = 50 (SCRBL + U18), *n* = 85 (CCAD), *n* = 59 (CCAD + U18), *n* = 33 (Rosc) and *n* = 29 (Rosc + U18) neurons were analyzed across 5 independent isolations. All error bars represent SEM. Statistical significance was calculated using a two-way ANOVA test in (**A**)–(**E**) and One sample *t* test and Wilcoxon test in (**F**). ns: not significant; *P < 0.05; **P < 0.01; ***P < 0.001; ****P < 0.0001. ^##^*P* < 0.01; ^#^ indicates comparison with the CTL condition. SCRBL is scramble, U18 is U18666A, CCAD is calcium channel association domain, Rosc is Roscovitine, TTX is tetrodotoxin, Nif is Nifedipine, XestoC is Xestospongin C.

The remodeling of ER–Mito Ca^2+^ handling proteins following loss of NPC1 function prompted us to ask if free Ca^2+^ levels within the ER and mitochondria are also altered in neurons. Previously it has been shown in non-excitable cell models of NPC disease, including cells harboring the most prevalent patient mutation, that loss of NPC1 function results in decreased ER Ca^2+^ (Ca^2+^_ER_) levels due to increased ER Ca^2+^ leak through IP_3_R1^17,23^. To test if similar results occur in neurons, we transfected neurons with the ER-targeted GCaMP indicator, GCaMP6-150^84^, and treated with vehicle control or U18. Aligned with published results, loss of NPC1 function resulted in about a 50 % decrease in GCaMP intensity (Fig. 10A). These data support published data that loss of NPC1 function decreases Ca^2+^_ER_.

In NPC disease, decreases in Ca^2+^_ER_ occur due to enhanced opening of IP_3_R1 receptors^17^. Given the enhanced proximity and size of IP_3_R1–VDAC1 interactions (Fig. 9C), we tested if leaky IP_3_R1 receptors result in elevated neuronal mitochondrial Ca^2+^ (Ca^2+^_Mito_). To evaluate Ca^2+^_Mito_ we transfected cortical neurons with a mitochondria-targeted RCaMP plasmid (pCAG-mito-RCaMP1h)^85^, and incubated neurons with vehicle or U18 for 24 h. Consistent with the model that leaky IP_3_R1 on ER membranes drive Ca^2+^ entry into mitochondria, normalized pCAG-mito-RCaMP1h intensities were significantly elevated following the loss of NPC1 function (Fig. 10B). To test a central role for ER Ca^2+^ stores and IP_3_R1 in facilitating increases in neuronal Ca^2+^_Mito_ we treated with vehicle or xestospongin C (XestoC; IP_3_R inhibitor) and found that U18-dependent elevations in Ca^2+^_Mito_ were abrogated (Fig. 10B). To further define the upstream source of Ca^2+^, we treated neurons with vehicle control and nifedipine (L-type Ca_V_1 inhibitor; Nif). Consistent with the model that Ca_V_1 channels play a central role in mediating increases in Ca^2+^_Mito_ in NPC disease, treatment with nifedipine abolished increases in Ca^2+^_Mito_ (Fig. 10B). Interestingly, AP blockage by TTX also prevented elevated Ca^2+^_Mito_ in U18 neurons (Fig. 10A), suggesting that increased neuronal excitability observed in NPC disease leads to elevated Ca^2+^_Mito_. Since increased K_V_2.1–Ca_V_1.2 interactions play a major role in Ca_V_1.2 clustering (Fig. 4) and in localized Ca^2+^ activity at K_V_2.1-forming ER–PM MCSs (Fig. 8), we predicted that K_V_2-dependent interactions with Ca_V_1 may underlie elevations in Ca^2+^_Mito_ in NPC disease. To test this prediction, we incubated neurons with CCAD to uncouple K_V_2 from Ca_V_1 and determined that like nifedipine treatment, disengaging K_V_2 from Ca_V_1 resulted in neuronal Ca^2+^_Mito_ being refractory to U18 (Fig. 10C). Similar results were observed with roscovitine (Fig. S9B) and place K_V_2.1–Ca_V_1 interactions at ER–PM MCSs as the most upstream source of Ca^2+^ driving increases in Ca^2+^_Mito_ in NPC disease. Alterations in Ca^2+^_Mito_ are often accompanied by alterations in reactive oxygen species (ROS) and changes in mitochondrial membrane potential (MMP) therefore we tested if they were also altered in models of NPC. Like Ca^2+^_Mito,_ both ROS and MMP were differentially changed following NPC1 inhibition with levels of ROS increased (Fig. 10D) and MMP less polarized (Fig. 10E). Significantly, treatment of neurons with CCAD rescued defects in ROS and MMP.

Sustained elevations in Ca^2+^_Mito_ or alterations in ROS and MMP can be detrimental to neuronal health and are frequently triggers for neurodegeneration. Thus, we tested if the mitochondrial phenotypes noted above correlate with changes in neuronal fidelity in NPC disease. As shown in Fig. 10F, viability was significantly decreased following 48 h of NPC1 inhibition. Treatment with XestoC or roscovitine abolished NPC1-driven toxicity (Fig. 10F) emphasizing the important roles IP_3_R and CDK5 play in decreasing neuron viability. Moreover, uncoupling K_V_2.1–Ca_V_1.2 interactions through the CCAD peptide completely rescued neurons from toxicity (Fig. 10F).

Collectively, these data suggest that augmented Ca_v_1.2–K_v_2.1 interactions at ER–PM MCSs drive deviant increases in Ca^2+^_Mito_ to promote neurotoxicity in NPC disease.

## Discussion

In the present study, we provide evidence linking the NPC1 cholesterol transport to the regulation of ER–PM and ER–Mito Ca^2+^ signaling nanodomains and neurodegeneration. We show in models of NPC disease that loss of NPC1 cholesterol transporter function facilitates a K_V_2.1-dependent reorganization of Ca_V_1.2–SERCA–RyR Ca^2+^ handling proteins to potentiate Ca^2+^ entry at ER–PM MCSs. In parallel with these changes, we also report the molecular remodeling of the IP_3_R1–GRP75–VDAC1 Ca^2+^ complexes at ER–Mito MCSs. Collectively, NPC1-dependent remodeling of these junctions creates a damaging feed-forward Ca^2+^ signaling axis which drives Ca^2+^ entry into mitochondria leading to neurotoxicity. Importantly, we show that uncoupling K_V_2–Ca_V_1 interactions at ER–PM MCSs rescues the NPC1-dependent mitochondrial Ca^2+^ defects and neurotoxicity, strongly suggesting that Ca^2+^ entry at upstream ER–PM MCSs is a key driver of Ca^2+^-dependent neurodegeneration (see Fig. S10 for model).

NPC disease is a neurodegenerative disorder that is characterized by the accumulation of cholesterol within the lysosome. In addition to this cellular phenotype, neurons from several models of NPC disease are hyperexcitable and fire APs at a higher frequency than WT neurons^52^. A simple prediction was that this hyperexcitability phenotype would increase voltage-dependent Ca^2+^ entry into NPC neurons. Indeed, we demonstrate that NPC1 loss of function leads to more frequent, electrically driven oscillations in intracellular Ca^2+^. In addition, we have also discovered that alterations in the nanoscale organization of Ca^2+^ handling proteins, localized at ER–PM MCSs, further potentiates Ca^2+^ entry. Central to these structural rearrangements is K_V_2.1 which influences the distribution and activity of Ca_V_1 channels, RyR, and SERCA^2,31,36,75^. We find that increased clustering of K_V_2.1 enhances interactions with Ca_V_1 channels to increase their density and cluster size. This is important as the distribution of Ca_V_1 channels dictates their function with clustered Ca_V_1 channels functionally communicate with one another via their C-termini to mediate their cooperative gating^57–61^. The opening of Ca_V_1 channels within a cluster is driven by the channel with the highest open probability leading to an overall enhancement in channel activity and resultant amplification of Ca^2+^ influx. Observations of modified Ca_V_1 channel nanoscale structure–activity relationships have been proposed as key contributors to the disease progression of pathologies such as Timothy syndrome^60^, hypertension^60^, and diabetes^86^, and here we detail similar observations in a neurodegenerative disorder. It is conceivable that similar changes in the nanoscale distribution and activity of Ca_V_1 channels may occur in other neurodegenerative disorders where dysfunctional Ca^2+^ signaling and altered cholesterol homeostasis have been observed^8,9,38,45,87–89^.

The molecular link between NPC1 and enhanced K_V_2.1–Ca_V_1 interactions appears to involve the protein kinase CDK5. Phosphorylation of K_V_2.1 by CDK5 enhances channel clustering^67^. Furthermore, CDK5 kinase activity is elevated in NPC disease^68,90^. We find that CDK5 activators, p35/p39 have increased expression in NPC disease and inhibition of CDK5 kinase activity with roscovitine abrogates enhancement of both K_V_2.1 and Ca_V_1.2 clustering in the PM of NPC neurons. Based on this information, we propose a model wherein loss of NPC function increases CDK5 partitioning to the plasma membrane resulting in a shift in the fractional amount of phosphorylated K_V_2.1, enhancing its clustering and consequently the distribution and activity of Ca_V_1 channels at the PM. Interestingly, a parallel consequence of increased phosphorylation of K_V_2.1 would be a decrease in its overall activity as a K^+^ conducting channel^91^ which may potentially contribute to the hyperexcitability phenotype in NPC^52^. Many questions arise from our findings, for example, what is the link between NPC1 and CDK5 activity? We provide evidence that hyperactive mTORC1 activity may be part of the molecular pathway between NPC1 and increased ER–PM Ca^2+^ activity in NPC but more experiments are required to understand the precise steps that allow such signaling to proceed. Second, with the increases in excitability and Ca^2+^ entry: why doesn’t calcineurin activity act as a homeostatic counterbalance to CDK5 to ensure K_V_2.1 clustering at ER–PM contact sites stays in a ‘physiological’ range? Perhaps the depleted lysosomal Ca^2+^ stores in NPC depress the catalytic activity of calcineurin^92,93^. We provide evidence that CDK5 and K_V_2.1 come into closer proximity following NPC1 inhibition which perhaps favors a net increase in K_V_2.1 phosphorylation and consequently K_V_2.1–Ca_V_1 complex formation. Future analyses should begin targeting these key questions to unmask and define the complete molecular cascade that exists between NPC1 cholesterol efflux and the tuning of ion channel distribution at ER–PM MCSs.

In addition to physically interacting to organize Ca_V_1 channels, K_V_2 also functionally coordinates RyR and SERCA at ER–PM nanodomains^2,31,35^. We find that loss of NPC1 function positively remodels the distribution of these proteins at ER–PM MCSs to increase Ca^2+^ entry into the cytoplasm. It is noteworthy that as K_V_2.1 cluster size and density increase so does the number of ER–PM MCSs. This observation aligns with reports that phosphorylated K_V_2.1 interacts with the ER VAP proteins to generate ER–PM MCSs^34,35^. However, our data suggest that it is not simply the physical coupling of K_V_2.1–VAP that defines this sub-population of ER–PM MCSs, as uncoupling Ca_V_1 from K_V_2 channels with CCAD, a perturbation that maintains K_V_2.1–VAP interactions^2^, decreases the overall number and density of ER–PM MCSs. That uncoupling of K_V_2–Ca_V_1 channels reduces the amount of Ca^2+^ entry into the cytoplasm (Fig. 8C and ref. ^2^) supports that K_V_2–Ca_V_1 dependent Ca^2+^ entry is either a stimulus for stabilizing ER–PM MCS or an instructive signal to induce the formation of new ER–PM MCSs in neurons. The latter model that Kv2–Ca_V_1 Ca^2+^ entry nucleates the formation of new MCSs aligns with previous reports that a number of lipid binding ER–PM MCS forming proteins are sensitive to local changes in cytoplasmic Ca^2+^ concentrations^29,94^ and provides evidence that K_V_2 channels are a crucial foundational element that regulates multiple sub-populations of ER–PM MCSs.

In the present study, we find that NPC1 deficiency leads to increased proximity between Ca_V_1.2–RyR and K_V_2.1–SERCA, suggesting that K_V_2.1-associated ER–PM MCSs are enriched in RyR, SERCA and Ca_V_1.2, consistent with previous proteomics analyses of Kv2.1-containing ER-PM MCSs purified from mouse brain^31^. Having more Ca_V_1.2 juxtaposed to RyR would amplify Ca^2+^ entry into the cytoplasm through Ca^2+^-Induced Ca^2+^-Release (CICR). Such amplification of Ca^2+^ signals is important in normal neuronal function as it facilitates excitation-transcription coupling^2^, however, in NPC disease it is further increased and together with the hyperexcitability phenotype results in aberrant and ultimately neurotoxic (see below) increases in Ca^2+^. An intuitive prediction of enhanced K_V_2–Ca_V_1–RyR Ca^2+^ entry in NPC disease would be an increase in ER Ca^2+^ levels. The statement is supported by the increase in clustering at ER–PM MCSs of two ER Ca^2+^ uptake mediator proteins, K_V_2.1 and SERCA (Fig. 3 and S7A)^75^, and increased SERCA activity in NPC disease models^23^. Thus, it was initially surprising that despite the alignment of these factors, ER Ca^2+^ levels are significantly decreased in NPC disease (Fig. 10A and ref. ^23^). The molecular mechanism underlying reduced ER Ca^2+^ in NPC disease is enhanced spontaneous Ca^2+^ release from IP_3_R1 in ER membranes^17^. Evidence herein suggests that heightened RyR CICR activity may also contribute to the leaky ER Ca^2+^ phenotype in NPC disease.

A common feature in neurodegenerative disorders is mitochondrial Ca^2+^ overload leading to mitochondrial membrane rupture and release of pro-apoptotic factors^17,77,95–98^. The IP_3_R–GRP75–VDAC1 signaling axis is a hotspot for Ca^2+^ flux from the ER towards the mitochondria. Our data provide evidence that ER Ca^2+^ stores are low whereas mitochondrial Ca^2+^ is significantly increased in NPC neurons. Furthermore, we find that increases in mitochondrial Ca^2+^ correlate with alterations in MMP, increased ROS generation, and cell death supporting the model that mitochondrial Ca^2+^ overload is a mechanism for neurodegeneration in NPC disease. Mechanistically, increased interactions between IP_3_R–GRP75–VDAC1 at ER–Mito MCSs coupled with more spontaneously active IP_3_R1^17^ creates the opportunity for destructive funneling of Ca^2+^ into mitochondria. We propose that in NPC disease, excess K_V_2–Ca_V_1 Ca^2+^ entry at ER–PM MCSs is transported into the ER through increased SERCA activity^23^ and clustering but instead of accumulating to increase ER Ca^2+^ concentrations, is quickly released by IP_3_R1 receptors at ER–Mito MCSs to promote neurotoxic increases in mitochondrial Ca^2+^ (Fig. S10). Supporting this model, we demonstrate that disrupting K_V_2–Ca_V_1 interaction or reducing CDK5 phosphorylation of K_V_2 channels reduces mitochondrial Ca^2+^ levels and rescues neuronal death following NPC1 inhibition, supporting the concept that K_V_2–Ca_V_1 Ca^2+^ entry at ER–PM MCSs is an upstream contributing factor to neurotoxic increases in mitochondrial Ca^2+^ in NPC. Future investigations should lead to a better understanding of whether disrupting Ca^2+^ entry at K_V_2–Ca_V_1 nanodomains has beneficial effects on disease progression in NPC disease models, as it does for stroke models^66,99^. Noteworthy, previous reports have linked altered K_V_2.1 activity to cell death through increased K^+^ efflux activating pro-apoptotic pathways^100,101^ rather than by favoring Ca^2+^ entry as we describe for NPC disease. Together, these data reinforce the need for homeostatic control of conducting (K^+^ efflux) and non-conducting (Ca^2+^ nanodomain organization) K_V_2.1 subpopulations by protein kinases and phosphatases to ensure the maintenance of neuronal health.

Why is there such a prominent Ca^2+^ phenotype across multiple organelles in NPC disease? Our working model is that alterations in Ca^2+^ gradients across MCSs represent cellular programs attempting to redistribute cholesterol rapidly and efficiently to cellular membranes to restore cholesterol homeostasis^93,102,103^. In this model, unless new homeostatic set points are found quickly, gross alterations in Ca^2+^ gradients, as well as lipids^16,52^, provide a substrate for neurodegeneration^17,23^. To design rational targets to slow or abrogate NPC-linked pathology, further quantitative characterization of the structural and functional alterations to ER–PM contacts and the downstream consequences linking mitochondrial Ca^2+^ elevations to neurodegeneration are required.

To conclude, this study details a molecular link between cholesterol egress from lysosomes and the reorganization of Ca^2+^ handling proteins at two membrane contact sites: ER–PM and ER–Mito. Our data demonstrate that NPC is a nanostructural ion channel clustering disease with altered ion channel distribution/activity at membrane contacts contributing to neurodegeneration.

## Methods

### Key resources

Key resources are documented in Supplementary Table 1.

### Animals

All experiments were performed in strict compliance with the University of California Davis ethical regulations for studies involving animals as approved by the University of California Davis Animal Care and Use Committee (protocol #: 22644). C57BL/6 WT and NPC1^I1061T^ mice were purchased from The Jackson Laboratory and kindly provided by Daniel Ory^64^, respectively. Mice were maintained under standard light-dark cycles, fed standard chow and water ad libitum, and housed in a vivarium with controlled conditions.

### Cell culture

tsA201 cells were purchased from Sigma (Cat #96121229), CHO WT and NPC1^−/−^ cells were a kind gift from Dr. Ory (Washington University, St. Louis, MO), HeLa WT and NPC1^−/−^ cells were a kind gift from Dr. Judith Storch (Rutgers). tsA201 and HeLa cells were cultured in DMEM supplemented with 10% FBS (GIBCO, Cat #26140-079), with 2 mM L-glutamine (GIBCO, Cat #25030-081) and 0.2% Penicillin/Streptomycin (GIBCO, Cat #15140-122). CHO cells were cultured in DMEM/F12 (1:1) (GIBCO, Cat #11320033) supplemented with 10% FBS (GIBCO, Cat #26140-079) and 0.2% Penicillin/Streptomycin (GIBCO, Cat #15140-122). All cell lines were passaged twice weekly and incubated in 5% CO_2_ at 37 °C. NPC1^−/−^

Cortical neurons from mice of both sexes were dissociated at embryonic day 15–18 (E15–18) using the Papain Dissociation System purchased from Worthington (Cat #LK003150). All stock solutions were prepared as per manufacturer’s recommendations. Dissection was conducted in sterile PBS at 4 °C as previously described in^104^ and meninges, cerebellum, hippocampus, and striatum were discarded. Cortical tissue was pelleted and incubated in papain for 20 min at 37 °C (agitating every 5 mins), followed by trituration. Consequently, cells were centrifuged 5′ at 1000*g* and resuspended in Earle’s Balanced Salt Solution (EBSS), ovomucoid (papain inhibitor) and DNase. Neurons were plated in Neurobasal (21103-049; Gibco) supplemented with B27 (GIBCO, Cat #17504-044), Glutamax (GIBCO, Cat #35050-061) and 0.2% penicillin/streptomycin at a density of 650,000 cells / mL. Neurons were incubated in 5% CO_2_ at 37 °C and half of the media was changed every 4 days. Experiments were performed at DIV 6-8, except for the GCaMP3-K_v_2.1_P404W_ Ca^2+^ imaging which was conducted at DIV 14–16. Rat cortical neurons were dissected and isolated at gestation day 18 as previously described in Vierra et al.^31^.

### Reagents

Xestospongin C (XestoC) was dissolved in DMSO and used at a final concentration of 10 µM and incubated overnight (O/N) or for 48 h. Torin-1 was dissolved in DMSO and used at a final concentration of 10 µM and incubated O/N. U18666A (U18) was dissolved in DMSO to a final concentration of 10 µM and incubated O/N or for 48 h. Tetrodotoxin (TTX) was dissolved in DMSO and incubated O/N at a final concentration of 200 nM. roscovitine (Rosc) was dissolved in DMSO and incubated O/N or for 48 h at a final concentration 10 µM. Ionomycin was dissolved in DMSO and used at a final concentration of 2.5 µM for ∼6 min. TAT-FFAT-HA (472-481) (FFAT), TAT-HA-C1aB (CCAD) and their respective control scramble peptides TAT-HA-C1aB-Scr and TAT-FFAT-HA (472-481)-Scr (SCBRL) were dissolved in molecular biology grade water and used at a final concentration of 1 µM for 48 h.

### Fillipin staining

Cells were washed with PBS and fixed in 4% paraformaldehyde (Electron Microscopy Sciences, Cat #15710) for 20 min at 21 °C. Cells were stained with fillipin (100 µg/ml in PBS) for 2 h at room temperature (RT, 21–23 °C). During the fillipin staining process cells were protected from light exposure. Cells were excited using a 405-nm LED and light collected using a Plan-Apochromat 63×/1.40 oil-immersion lens and a Zeiss LSM 880 Airyscan microscope. Images were acquired in PBS solution at RT using Zen v2.3 software.

### Protein extraction and abundance determination

Protein was harvested from neuronal cultures in RIPA buffer (Thermo Scientific, Cat #89900) containing Complete/Mini/EDTA-free protease inhibitor cocktail (Roche, Cat #11836170001), sodium fluoride 1 mM (Sigma-Aldrich, Cat #67414), SDS 0.05%, sodium deoxycholate 0.4% (DOC) and Microcystin 4 µg/mL (Sigma Millipore, Cat #475821). Cells were scraped in 100 μL of lysis buffer and centrifuged at 13,200 rpm for 20 min at 4 °C to isolate the postnuclear supernatant. Protein concentration was quantified using a Pierce BCA protein assay kit (Thermo Scientific, Cat #23225). 15 μg of each protein sample was added to 4%–12% Bis-Tris gels and electrophoresed under reducing conditions for approximately 75 min at 155 V. Proteins were transferred onto nitrocellulose membranes (Life Technologies, Cat #LC2000) using the Mini-Blot system (Thermo Scientific, Cat #A25977) for 2 h 30 min at 12 V (∼200 mA). After 30 min incubation at RT in Tris-Buffered Saline (TBS) buffer supplemented with 0.05% Tween‐20 (TBS-T) and 5% non‐fat dry milk, membranes were exposed O/N at 4 °C to the following primary antibodies: K_V_2.1 at 4 μg/mL (NeuroMab, K89/34), Ca_V_1.2 at 1:250 (Alomone Labs, acc-003), CDK5 at 1:100 (Santa Cruz Biotechnology, Cat #sc-6247), p35 at 1:100 (Cell Signaling Technology, Cat #C64B10), p39 at 1:100 (Santa Cruz Biotechnology, Cat #sc-365781) or GAPDH at 1:1000 (Proteintech, Cat #10494-I-AP). Blot bands were detected using fluorescent secondary antibodies goat anti-rabbit 680RD at 1:1000 (LI-COR, Cat #P/N 926-68071) and goat anti-Mouse 800CW at 1:1000 (LI-COR, Cat #P/N 925-32210). Signals were detected using Azure Sapphire^TM^ Biomolecular Imager (Azure Biosystems) and quantified using the ImageJ software. Abundance of proteins was normalized to GAPDH.

### Transfection and plasmids

Plasmid transfection mixtures were prepared using 2.5 µl/ µg DNA of Lipofectamine 2000 (Invitrogen, Cat #11668-019), added to neurons and incubated for 2 days. The following plasmids (per 650,000 neurons) were used in this study: 4 µg pCAG-mito-RCaMP1h (Addgene, Cat #105013), 4 µg ER-GCaMP6-150 (Addgene, Cat #86918), 2 ug ArcLight-Q239 (Addgene, Cat #36856)^76^, 2 µg GFP-MAPPER^74^, 1 µg SPLICS_L_-P2A^ER-PM^ (Addgene, Cat #164111), 1 µg SPLICS_S_-P2A^ER-PM^ (Addgene, Cat #164112) and 1.1 µg GCaMP3-Kv2.1_P4O4W_^31^. Plasmid transfection mixtures for CHO cells were prepared using 7 µl of Lipofectamine LTX and 7 µl of Plus (Invitrogen, Cat #15338-030) and were imaged the day after transfection. The following plasmids were used: 1 µg per 960,000 cells rat Ca_V_1.2α1c (GenBank number NP_001129994.1; a gift from Dr. William Catterall, University of Washington, Seattle, WA), 1 µg per 960,000 cells rat Ca_V_α_2_δ (AF286488; a gift from Dr. Diane Lipscombe, Brown University, Providence, RI), 1 µg per 960,000 cells Ca_V_β_3_ (M88751; a gift from Dr. Diane Lipscombe, Brown University, Providence, RI) and 1 µg per 960,000 cells DsRed-K_V_2.1^35^.

### Super resolution airyscan imaging

Imaging was conducted at RT using a Zeiss LSM880 confocal laser scanning microscope equipped with a Plan‐Apochromat 63× 1.4 Oil DIC M27 objective and a super‐resolution Airyscan detection unit. Images of GFP/Alexa‐488, Alexa-568/mCherry and Alexa‐647 were collected using 488-, 594- and 633-nm excitation lasers, respectively, with multicolor images sequentially acquired. Fixed and live cells were acquired in PBS or Ringer’s solution (in mM, 160 NaCl, 2.5 KCl, 2 CaCl_2_, 1 MgCl_2_, 10 HEPES, and 8 D‐Glucose) with the ZEN software v2.3, respectively. Images were taken at 0.5 μm depth intervals within the cell (see PLA assay and ER staining in live neurons) or as a single internal or plasma membrane plane for HA, GRP75, IP_3_R and VDAC1, or, Ca_V_1.2, Ca_V_1.3, K_V_2.1, Ca_V_2.1 and RyR immunolabeling, respectively. 3D analysis for the ER morphology in neurons was conducted with the IMARIS software (Oxford Instruments); all other datasets were carried out following background subtraction and similar thresholding using ImageJ (NIH, Bethesda, MD, USA). Colocalization/overlap analyses were performed by multiplying binary masks of Ca_V_1.2, K_V_2.1, RyR, IP_3_R, VDAC1, ER and mitochondria images. For neuronal analyses, the soma area and a 15 µm^2^ ROI from proximal neuronal dendrites were used. For protein clustering and ER–PM MAPPER puncta studies in the soma and cell body, sum intensity and numbers of clusters were normalized to their respective areas.

### Super resolution TIRF imaging

Images were acquired using a Leica Infinity TIRF microscope equipped with a 163× 1.49 (fixed cells) and a 100× 1.47 (live cells) CORR TIRF oil immersion objective and a Hamamatsu orca flash 4.0 camera. Leica LAS X software was employed for both image acquisition and processing. GFP/YFP, Alexa‐647 and Alexa-568 images were collected with the 488 nm, 638 nm and 561 nm excitation lines, respectively. Fixed or live cells were imaged in a GLOX–MEA (Tris 10 mM pH8, Glucose Oxidase 56 mg/mL, Catalase 34 mg/mL, 10 mM MEA) or Ringer’s solution (in mM, 160 NaCl, 2.5 KCl, 2 CaCl_2_, 1 MgCl_2_, 10 HEPES, and 8 D‐Glucose), respectively. For fixed cell imaging (K_V_2.1–VAPA/B, K_V_2.1–CDK5, Ca_V_1.2, K_V_2.1, K_V_2.1–Ca_V_1.2 and K_V_2.1–SERCA) 50,000 cycles with an exposure time of 10 ms were acquired and imaging analysis was performed using IMARIS software (Oxford Instruments). For all analyses, cluster number was normalized to the respective soma area. Colocalization/overlapping analyses were performed by creating a 2D K_V_2.1 mask and measuring Ca_V_1.2, CDK5, VAPA/B or SERCA areas within said mask. Live cell imaging of GCaMP3-K_V_2.1_P4O4W_ analyses were performed using the ImageJ and ClampFit (Molecular Devices) softwares.

### Immunofluorescence immunocytochemistry

Antibody dilutions/amounts, validation, company names, catalogue numbers and clone numbers can be found either below, in the reporting summary, and/or supplementary table 1. Cells were fixed for 20 min at RT in PBS with 4% formaldehyde freshly prepared from paraformaldehyde and blocked for 1 h in 50% SEA BLOCK and 0.5% Triton X‐100 solution. Neurons were incubated O/N at 4 °C in 20% SEA BLOCK and 0.5% Triton X‐100 in PBS with the following primary antibodies: GRP75 1:100 (Abcam, Cat #ab2799), K_V_2.1 10 µg/mL (NeuroMab, K89/34), Ca_V_1.2 1:333 (Alomone, Cat #acc-003), K_V_2.1(pS603) 1:5 (L61/14.2), VDAC1 1:100 (Abcam, Cat #ab14734), SERCA 10 µg/mL (Abcam, Cat #ab2861), Ca_V_1.3 10 µg/mL (Alomone Labs, Cat #ACC-005), pan-RyR 1:100 (Abcam, Cat #ab2868), IP_3_R 18 µg/mL (Abcam, Cat #ab5804), Ca_V_2.1 1:200 (Alomone Labs, Cat #ACC-001), and K_V_2.1 pS603 1:5 (L61/14). After primary antibody incubation, neurons were washed 3 × 5 min and subsequently incubated for 1 h at RT with the following secondary antibodies: Goat anti-Mouse-647 and −568 nm 1:1000 (Invitrogen, Cat #A21236 and Cat #A11031, respectively), anti-Rabbit-647 and −555 nm 1:1000 (Invitrogen, Cat #A21245 and Cat #A21429, respectively), anti-Mouse IgG1-568 nm 1:1000 (Invitrogen, Cat #A21124) and anti-Mouse IgG1-CF568 1:250 (Sigma-Aldrich, Cat #SAB4600314).

### Immunofluorescent labeling of brain sections

*Fixation:* Postnatal day 60 (P60) wild-type and NPC1^I1016T^ mice were anesthetized and transcardially perfused with 4% phosphate buffered (PB) formaldehyde (pH 7.4) made from powdered paraformaldehyde using a peristaltic pump. Perfused brains were collected and cryoprotected with 30% sucrose/PB solution. *Sectioning*: Sagittal brains sections (30 µM) were generated using a freezing microtome and immunolabeling was performed on free floating sections. Briefly, tissue was blocked in 10% normal goat serum (NGS) with 0.3% TritonX-100 in 0.1 M PB at RT for 1 h followed by incubation in primary antibody O/N at 4 °C. Primary antibody incubation was performed in 5% NGS/0.3% TritinX-100/0.1 M PB. *Antibodies:* the following primary antibodies were used for immunolabeling: Calbindin 1:200 (Sigma-Aldrich #C9848),Calbindin, 2 µg/mL (L109/57, NeuroMab), K_V_2.1 10 µg/mL (NeuroMab, K89/34), Ca_V_1.2 1:200 (Alomone Labs, Cat #acc-003), Ca_V_1.2 5 µg/mL (NeuroMab, N263/31 R). Species-specific Alexa Fluor 488, 555 and 647conjugated secondary antibodies (2 µg/mL; Life Technologies) were incubated for 60 min at RT. Images were taken using a Zeiss ApoTome Fluoview FV3000 confocal microscope (Olympus) or a Zeiss Axioscope 2 widefield microscope with ApoTome. All images were assembled using FIJI and Illustrator (Adobe). Ca_V_1.2–K_V_2.1 colocalization volumes were calculated using IMARIS software by quantifying the K_V_2.1-positive pixels inside a 3D Ca_V_1.2 mask.

### PLA assay

The Duolink In Situ PLA kit (Sigma-Aldrich, Cat #DUO92004-100RXN and DUO92002-100RXN) was used to quantify Ca_V_1.2-K_V_2.1 proximity in fixed cortical neurons treated with U18 or vehicle control (DMSO). Following fixation, as described in *Immunofluorescence Immunocytochemistry* above, cells were incubated for 15 min with 100 mM glycine at RT. Subsequently, neurons were washed in PBS (2 ×3 min) and permeabilized in 0.1% Triton-X100 for 20’ at RT. Neurons were blocked in a 20% SEA BLOCK solution (Thermo Scientific, Cat #37527) for 1 h and incubated at 4 °C O/N with the following primary antibodies: K_V_2.1 10 µg/mL (NeuroMab, K89/34) and Ca_V_1.2 1:333 (Alomone, Cat #acc-003;1:333). Secondary oligonucleotide-conjugated antibodies (PLA probes: anti-mouse MINUS and anti-rabbit PLUS) were used at 1:5 dilution in Duolink Antibody Diluent. PLA assay was performed as per the manufacturer’s recommendations. Coverslips were mounted with DAPI Fluoromount-G (SouthernBiotech, Cat #0100-20). The fluorescence signal was detected using a Zeiss LSM880 confocal laser scanning microscope equipped with a Plan‐Apochromat 63× 1.4 Oil DIC M27 objective and a super‐resolution Airyscan detection unit. 405- and 488-nm lasers were employed to visualize the DAPI-stained nucleus and PLA signal, respectively. For each neuron, optical *z*-axis sections were acquired at 0.5 μm intervals and combined to a single maximum intensity projection using ImageJ.

### ER and mitochondrial staining in live neurons and tsA201 cells

Cells were incubated for 20 min using Mito Tracker (50 nM; Invitrogen, Cat #M22426) and ER Tracker (100 nM; Invitrogen, Cat #E12353). Cells were subsequently washed twice (5 min each time) before being imaged with a Zeiss LSM880 confocal laser scanning microscope equipped with a Plan‐Apochromat 63× 1.4 Oil DIC M27 objective and a super‐resolution Airyscan detection unit; 633- and 546-nm excitation lasers were used to image mitochondria and ER, respectively. For tSA201, cells single planes near the PM were acquired and overlapping areas between both organelles were analyzed by multiplying their respective binary masks in ImageJ and normalized to the respective cell body area. For neurons, z-stacks were taken at 0.5 μm depth intervals within the cell and analysis was performed in the IMARIS software (Oxford instruments).

### Mitochondrial and ER Ca^2+^ imaging

48 h post-transfection with pCAG-mito-RCamPh1 or ER-GCaMP6-150, neurons were imaged in a 2 mM Ca^2+^ Ringer’s solution using a LSM880 confocal laser scanning microscope equipped with a Plan‐Apochromat 63× 1.4 Oil DIC M27 objective and a super‐resolution Airyscan detection unit. A 546 nm laser was used to excite pCAG-mito-RCamPh1, while a 488 nm laser was used to excite ER-GCaMP6-150. After collection of a baseline image, cells were perfused with a 20 mM Ringer’s solution containing 2.5 µM Ionomycin for 6 min. The mean intensity ratio of pre-ionomycin/post-ionomycin signals was calculated for each cell. Higher ratios were taken to indicate higher resting mitochondrial or ER Ca^2+^ concentrations.

### Cytosolic Ca^2+^ imaging

Cells were incubated in a 2 mM Ca^2+^ Ringer’s solution containing 2.5 µM Fluo-4 and 0.1% Pluronic acid for 30 min. Cells were then moved to a Fluo-4–free Ringer’s solution to deesterify the Fluo-4 for 30 min. Following deesterification, Fluo-4–loaded cells were excited with a 488-nm laser and the resulting fluorescence monitored using an inverted microscope with a Plan-Apochromat 63×/1.40 oil objective, connected to an Andor W1 spinning-disk confocal with a Photometrics Prime 95B camera. Images were acquired every 50 ms for a total of 50 s in a 2 mM Ca^2+^ Ringer’s solution at RT using Micromanager (1.4.21) software. Electrical stimulation at 40 V was performed at 1 Hz for 5 s with a Field Stimulator (IonOptix). Intracellular Ca^2+^ activity was measured as Ca^2+^ peak frequency and Ca^2+^ peak amplitude in a Region of Interest (ROI) localized in the soma or proximal neuronal dendrites using ImageJ or ClamPfit software.

### GCaMP3-K_v_2.1_P4O4W_ imaging

48 h post-transfection, TIRF imaging was performed using a Leica Infinity TIRF super-resolution microscope equipped with a 100× 1.47 TIRF oil immersion objective and a Hamamatsu Orca Flash 4.0 camera. Images were acquired in Ringer’s solution (in mM, 160 NaCl, 2.5 KCl, 2 CaCl_2_, 1 MgCl_2_, 10 HEPES, and 8 D‐Glucose) containing B-Kay 500 nM every 100 ms for a total time of 50–100 s by exciting with a 488-nm laser. Ca^2+^ activity was measured as *GCaMP3-K*_*v*_*2.1*_*P4O4W*_ peak amplitude in a *ROI* localized in the soma or proximal neuronal projections using ImageJ and ClamPfit softwares.

### H2DCFDA assay (ROS production)

The ROS production assay was conducted as per the manufacturer’s recommendations (Invitrogen, Cat #D399). Live DIV 6-8 cortical neurons were incubated O/N with U18 and CCAD or SCRBL peptides. Single-plane images were collected in Ringer’s solution (in mM, 160 NaCl, 2.5 KCl, 2 CaCl_2_, 1 MgCl_2_, 10 HEPES, and 8 D‐Glucose) at 488 nm using a Plan‐Apochromat 63× 1.4 Oil DIC M27 objective and a Zeiss 880 Airyscan microscope at RT. Images were analyzed using ImageJ and mean intensities were taken to plot ROS production.

### *MitoProbe JC-1 assay* (mitochondrial membrane potential)

Mitochondrial membrane potential was conducted as per the manufacturer’s recommendations (Abcam, Cat #ab113850) and plotted as a ratio of aggregated JC-1 form/monomer JC-1 form, with higher ratios indicating more polarized membranes. JC-1 aggregates were imaged at a 618 nm emission and a 488 nm excitation, and JC-1 monomers at a 536 nm emission and 488 nm excitation in Ringer’s solution (in mM, 160 NaCl, 2.5 KCl, 2 CaCl_2_, 1 MgCl_2_, 10 HEPES, and 8 D‐Glucose) using a Plan‐Apochromat 63× 1.4 Oil DIC M27 objective and a Zeiss 880 Airyscan microscope at RT. Images were analyzed using ImageJ software.

### Cell viability assay

The cell viability assay was conducted as per the manufacturer’s recommendations (K502-100; BioVision). Live DIV 6–8 cortical neurons were incubated 48 h with U18, XestoC, Rosc or CCAD or SCRBL peptides before being washed once with 2 mM Ca^2+^ Ringer’s solution and loaded with 1 mL assay buffer containing 2 µL Live cell staining dye and 1 µL Dead cell staining dye. Cells were immediately excited using a 488- and 564- nm LED. Single-plane images were collected using a Plan‐Apochromat 63× 1.4 Oil DIC M27 objective and a Zeiss 880 Airyscan microscope at RT. Images were analyzed using ImageJ. The ratio of live (488 nm) to dead (564 nm) was taken for each single neuron every captured image, with higher ratios indicating increased neuronal viability.

### Statistics and reproducibility

The number of replicates is based on previously published observations to reached statistical differences between datasets^16,17,23,26,52,104–107^. When comparing two or three independent groups, normality was determined with the Agostino-Pearson omnibus test and a parametric, unpaired *t* test, or nonparametric, Mann–Whitney *t* test, was employed. When comparing the means of 4 groups with two or more categorical independent variables, the two-way ANOVA for multiple comparisons with appropriate post hoc test was used. When quantifying fold changes respective to the control condition, one sample *t* test was employed. *P* < 0.05 was considered statistically significant (*P ≤ 0.05, **P ≤ 0.01, ***P ≤ 0.001, ****P ≤ 0.001); # shows significance between sample groups and the negative control when ANOVA was employed. To determine whether values within a dataset have significant outliers, a two‐sided Grubbs’ test, with an alpha value of 0.05 was performed. All the data values are presented as means ± SEM and the number of technical and biological replicates is detailed in each figure legend, which is based on previously published observations and is consistent with the general number of replicates and conditions commonly accepted in the field. For each neuronal dataset experiments were performed from 1-4 independent neuronal cultures with each isolation containing 6–8 pups. Several key datasets were blinded and outcome accessed.

### Reporting summary

Further information on research design is available in the Nature Portfolio Reporting Summary linked to this article.

## Supplementary information

Supplementary information

Peer Review File

Description of Additional Supplementary Files

Supplementary Movie 1

Supplementary Movie 2

Reporting Summary

## Data Availability

The data supporting the findings of this work can be found in the source data file and additional raw data will be made available upon request. Source data are provided with this paper.

## Source data

Source Data
