# Supplementary material for: NPC1-dependent alterations in KV2.1–CaV1.2 nanodomains drive neuronal death in models of Niemann-Pick Type C disease
Source: Nat Commun. 2023 Jul 28;14:4553. doi: 10.1038/s41467-023-39937-w (PMC10382591; doi:10.1038/s41467-023-39937-w)

Supplementary information

NPC1-dependent alterations in $K_V2.1–Ca_V1$ nanodomains drive neuronal death

Maria Casas[1], Karl D. Murray[1,2], Keiko Hino[3], Nicholas C. Vierra[1], Sergi Simó[3], James S. Trimmer[1], Rose E. Dixon[1], and Eamonn J. Dickson[1]*.

Information within this file:

Figure S1. NPC1 loss of function impairs cholesterol distribution and voltage-dependent Ca^{2+} entry in neurons

Figure S2. NPC1 loss of function leads to decreased PM $Ca_V2.1$ cluster size and intensity.

Figure S3. NPC1 inhibition does not alter $Ca_V1.2$ or $K_V2.1$ total protein levels but does increase $K_V2.1$ clustering in the $NPC1^{I1061T}$ hippocampus.

Figure S4. Disrupting the $Ca_V1.2$-$K_V2.1$ interaction in NPC1 inhibited and NPC1 knock-out cells, abrogates the NPC1-dependent $Ca_V1.2$ increased clustering.

Figure S5. Targeting $Ca_V1.2–K_V2.1$ interactions and mTOR inhibition rescues $Ca_V1.2$ distribution in rat neurons and impairs $Ca_V1.2–K_V2.1$ clustering.

Figure S6. Gross ER morphology is not affected by NPC1 inhibition.

Figure S7. NPC1 dysfunction enhances SERCA–$K_V2.1$ clustering and PM excitability.

Figure S8. NPC1 modulates the nano-distribution of ER - mitochondrial junctions without altering its gross structure.

Figure S9. NPC1 regulates mitochondrial Ca^{2+} levels and cellular viability.

Figure S10. NPC1 loss of function remodels ER–PM and ER–Mito membrane contacts to promote Ca^{2+}_{Mito}-driven neurotoxicity.

Supplementary Table 1: Resources table

Uncropped blots.

Figure S1

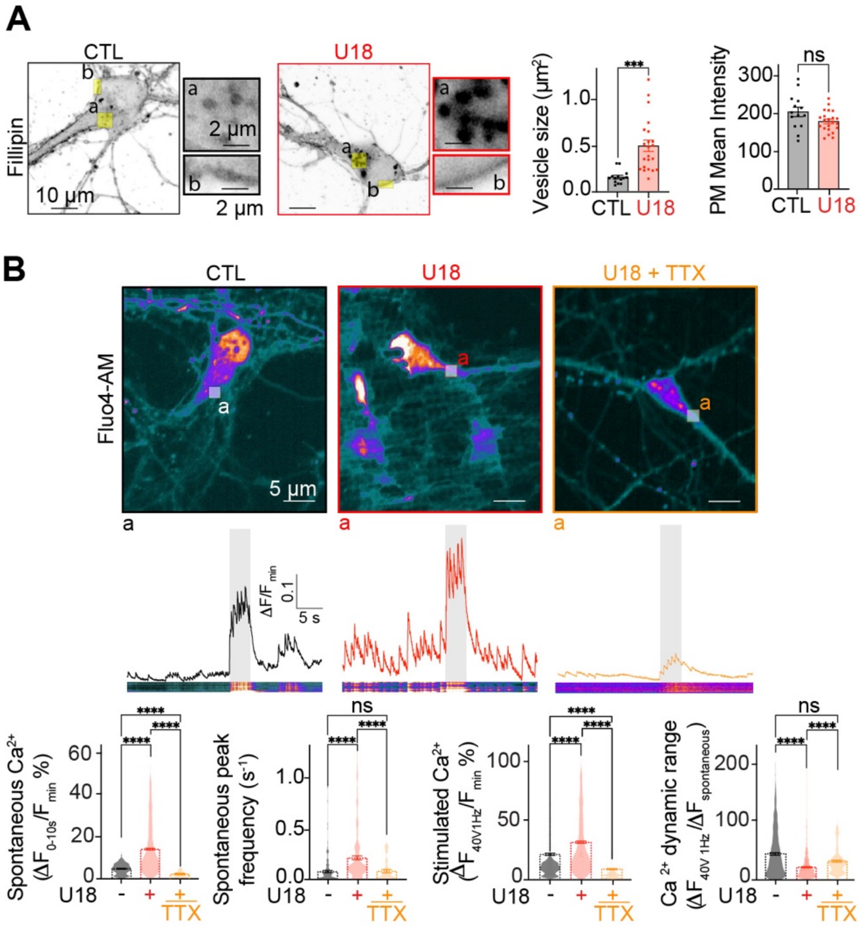

Figure S1. NPC1 loss of function impairs cholesterol distribution and voltage-dependent Ca^{2+} entry in neurons.

A) *Left*, representative super-resolution Airyscan images of free cholesterol in CTL (black) and U18 (red) neurons stained with Fillipin. *Right*, quantification of intracellular free-cholesterol vesicle size and free-cholesterol mean value in the PM of CTL (black) and U18 (red) neurons. N= 14 (CTL) and n= 22 (U18) neurons were analyzed across 1 isolation. **B**) *Top*, representative images and intracellular Ca^{2+} activity in electrically stimulated CTL (black), U18 (red) and TTX (orange) neurons. Electrically stimulation was performed from seconds 26 to 31 at 40V 1Hz. *Bottom*, quantification of spontaneous intracellular Ca^{2+} frequency, spontaneous and stimulated Ca^{2+} peak amplitudes, and Ca^{2+} dynamic range. N= 108 (CTL), n= 148 (U18) and n= 35 (TTX+U18) neurons; n= 138 (CTL), n= 683 (U18) and n= 60 (TTX+U18) spontaneous Ca^{2+} peaks and n= 406 - 457

(CTL), n= 656 - 707 (U18) and n= 162 (TTX+U18) electrically evoked Ca^{2+} peaks were analyzed across 1 isolation. All error bars represent SEM. Statistical significance was calculated using the following tests: unpaired *t*-tests (two-tail) in A) and Mann-Whitney *t*-test (two-tail) in B). ns: not significant; **P* < 0.05; ***P* < 0.01; ****P* < 0.001; *****P* < 0.0001. CTL is control, U18 is U18666A, and TTX is tetrodotoxin.

Figure S2

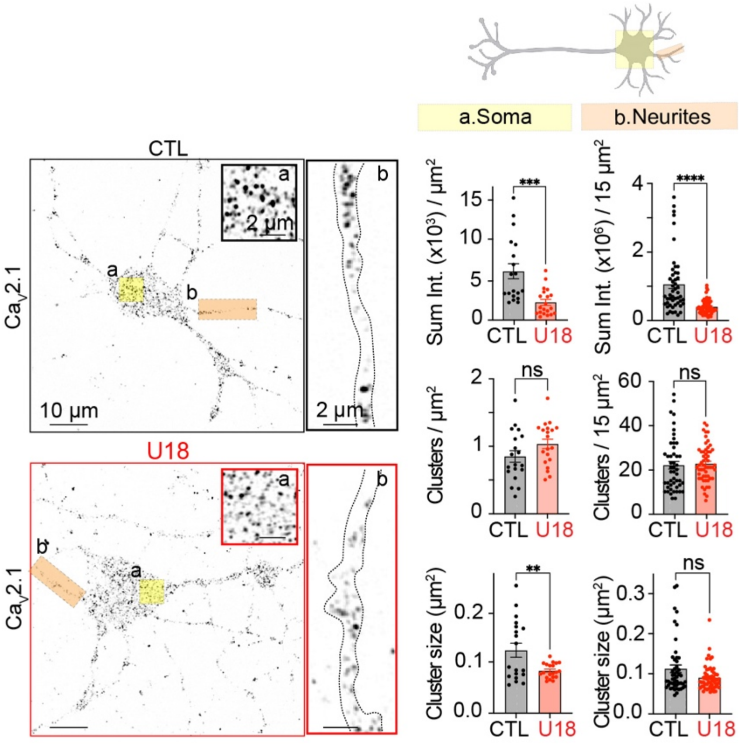

Figure S2. NPC1 loss of function leads to decreased PM $Ca_V2.1$ cluster size and intensity.

Left, representative super-resolution Airyscan images taken at a focal plane near the PM of CTL (black) and U18 (red) neurons immunolabeled for $Ca_V2.1$. *Right*, quantification of PM $Ca_V2.1$ total intensity, cluster density and cluster size in the soma (left, yellow) and dendrite (right, orange) regions of CTL (black) and U18 (red) neurons. N= 19 (CTL) and n= 20 (U18) neurons, and n= 49 (CTL) and n= 54 (U18) dendrites were analyzed across 2 independent isolations. All error bars represent SEM. Statistical significance was calculated using Mann-Whitney (two-tail) and Unpaired *t*-tests (two-tail). ns: not significant; *P < 0.05; **P < 0.01; ***P < 0.001; ****P < 0.0001. CTL is control and U18 is U18666A.

Figure S3

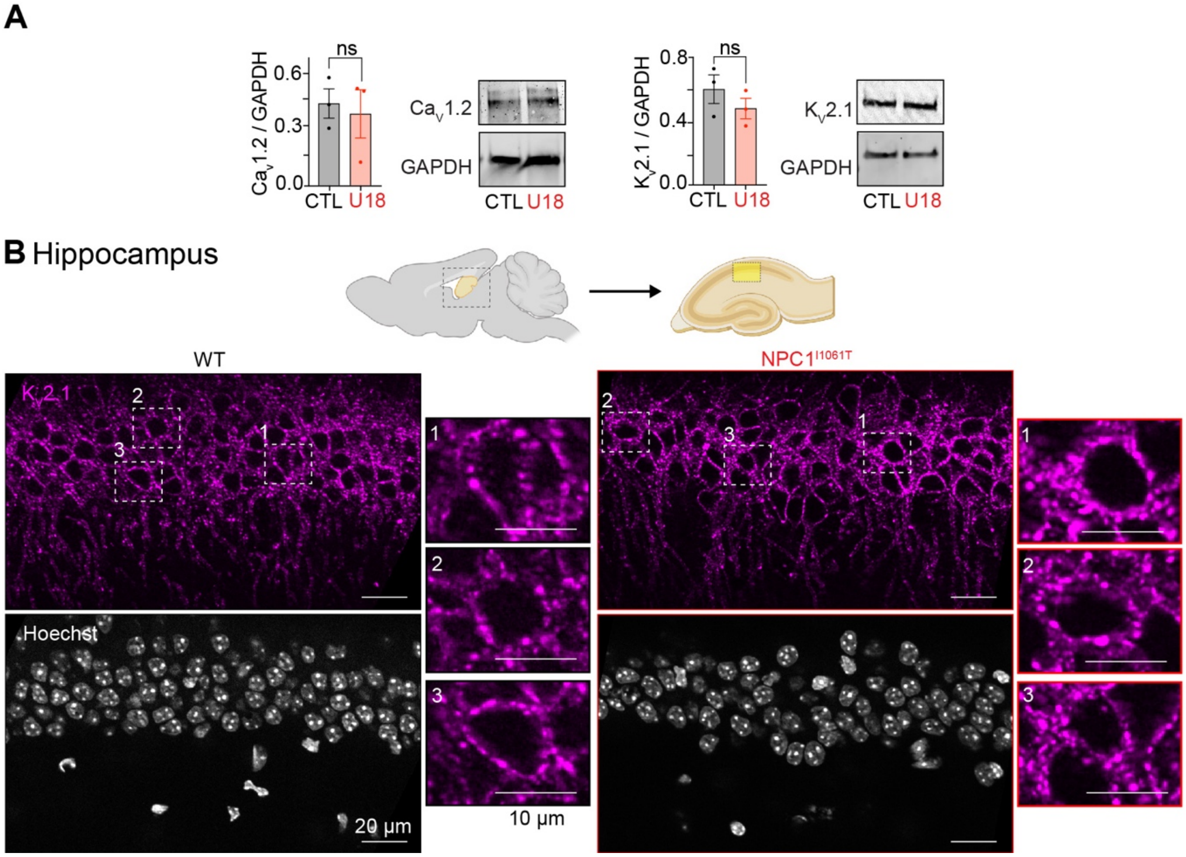

Figure S3. NPC1 inhibition does not alter Ca$_V$1.2 or K$_V$2.1 total protein levels but does increase K$_V$2.1 clustering in the NPC1^{I1061T} hippocampus.

A) *Top*, quantification of total Ca$_V$1.2 and K$_V$2.1 protein levels normalized to total GAPDH levels in CTL (black) and U18-treated (red) cortical neurons. N= 3 independent protein pools were analyzed from independent isolations. **B**) Representative hippocampal section from a WT (left-black) and NPC1^{I1061T} (right-red) mice fixed and immunolabelled for K$_V$2.1. This experiment was repeated across 2 pairs of animals. All error bars represent SEM. Statistical significance was calculated using Mann-Whitney (two-tail). ns: not significant. CTL is control, WT is wild-type, and U18 is U18666A.

Figure S4

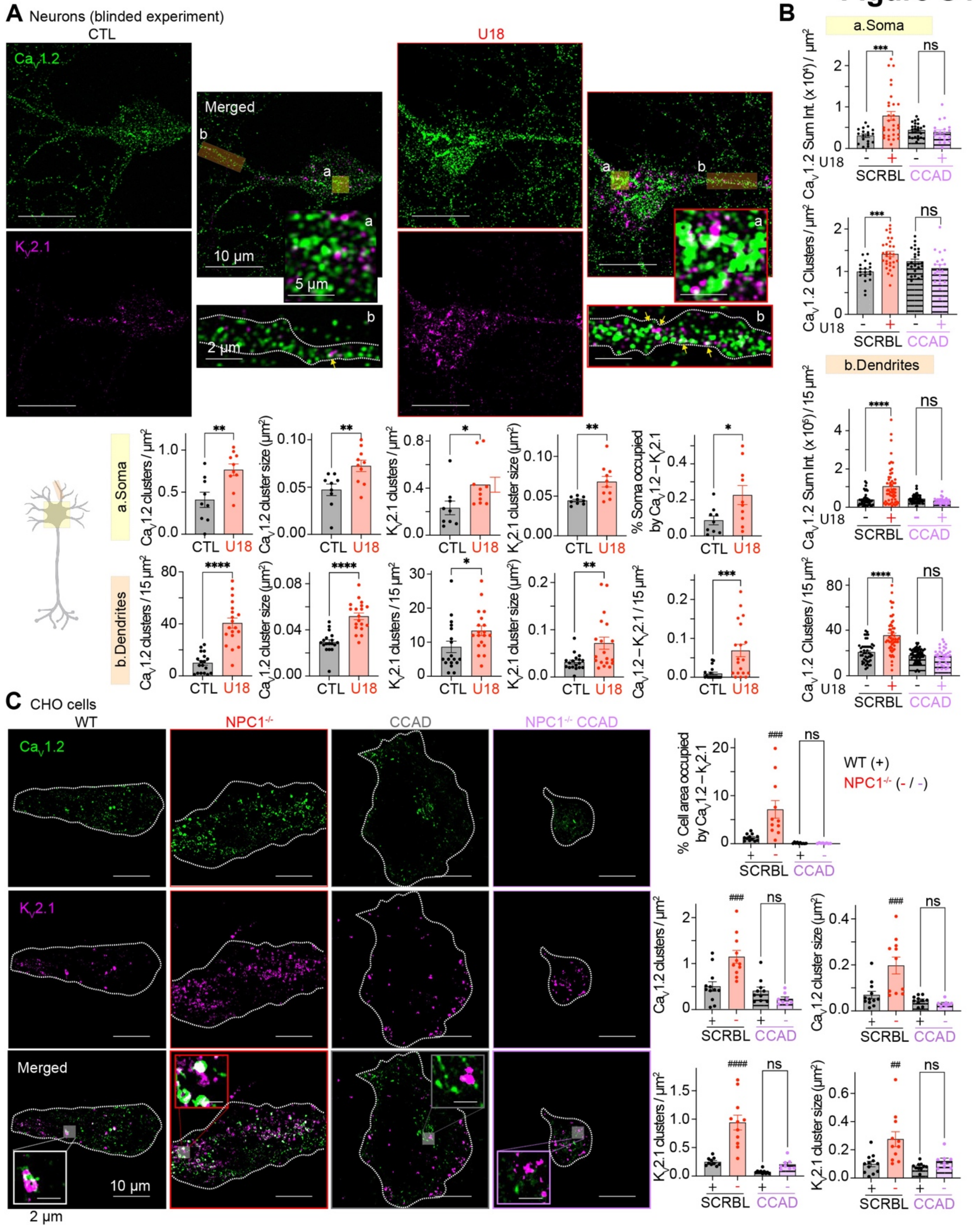

Figure S4. Disrupting the $Ca_V1.2$-$K_V2.1$ interaction in NPC1 inhibited and NPC1 knock-out cells, abrogates the NPC1-dependent $Ca_V1.2$ increased clustering.

A) Blinded experiments. *Top*, representative super-resolution Airyscan images taken at a focal plane near the PM of CTL (black) and U18 (red) neurons co-immunolabeled for $Ca_V1.2$ and $K_V2.1$. *Bottom*, quantification of $Ca_V1.2$, $K_V2.1$ and $Ca_V1.2$ – $K_V2.1$ clustering in the soma (top, yellow) and dendrite (bottom, orange) regions of CTL (black) and U18 (red) neurons. N= 9 and n= 10 neurons, and n= 18 and n= 19 dendrites were analyzed across 1 independent isolation. **B**) Quantification of PM $Ca_V1.2$ total intensity and cluster density of CTL (black) and U18-treated (red) neurons co-incubated with CCAD or SCRBL peptide (purple) in the soma (top, yellow) and dendrite (bottom, orange) regions. N= 18 (SCRBL), n= 30 (SCRBL+U18), n= 25 (CCAD) and n= 19 (CCAD + U18) neurons and n= 46 (SCRBL), n= 55 (SCRBL+U18), n= 57 (CCAD) and n= 34 (CCAD + U18) dendrites were analyzed across 1 independent isolation. **C**) Blinded experiment. *Left*, representative super-resolution Airyscan images taken at a focal plane near the PM of CTL (black) and $NPC1^{-/-}$ (red) cells co-transfected with a $Ca_V1.2$ and $K_V2.1$ plasmids and co-immunolabeled for $Ca_V1.2$ and $K_V2.1$. Right, quantification $Ca_V1.2$, $K_V2.1$ and $Ca_V1.2$ – $K_V2.1$ clustering of WT (black) and $NPC1^{-/-}$ (red) cells incubated with CCAD or SCRBL peptide (purple). N= 10 – 12 (SCRBL), n= 11 (SCRBL, $NPC1^{-/-}$), n= 10 (CCAD) and n= 8 (CCAD, $NPC1^{-/-}$) cells were analyzed across 2 independent cultures. All error bars represent SEM. Statistical significance was calculated using Mann-Whitney and unpaired t-tests in A) and two-way ANOVA test in B) and C). ns: not significant; $^{*}P < 0.05$; $^{**}P < 0.01$; $^{***}P < 0.001$; $^{****}P < 0.0001$. $^{\#}P < 0.05$; $^{\#\#}P < 0.01$; $^{\#\#\#\#}P < 0.0001$. $^{\#}$ indicates comparison with the CTL condition. CTL is control, SCRBL is scramble, U18 is U18666A, and CCAD is calcium channel association domain.

Figure S5

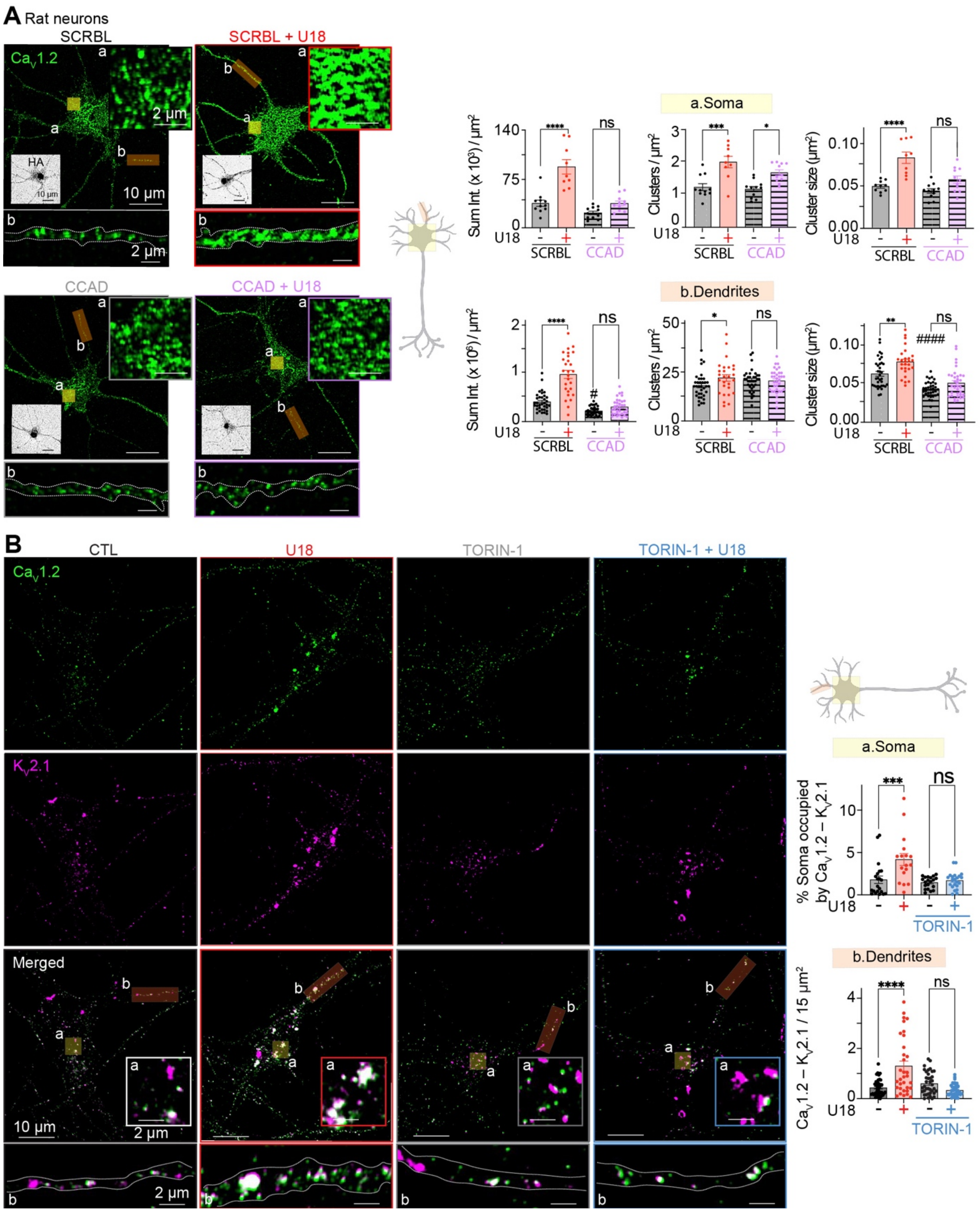

Figure S5. Targeting $Ca_V1.2$–$K_V2.1$ interactions and mTOR inhibition rescues $Ca_V1.2$ distribution in rat neurons and impairs $Ca_V1.2$–$K_V2.1$ clustering.

A) *Top*, representative super-resolution Airyscan images taken at a focal plane near the PM of CTL (black) and U18-treated (red) rat neurons co-incubated with an SCRBL or the CCAD peptide and immunolabeled for $Ca_V1.2$. *Bottom*, quantification of PM $Ca_v1.2$ total intensity, cluster density and cluster size in the soma (top, yellow) and dendrite (bottom, orange) regions of CTL (black) and U18 (red) rat neurons incubated with the CCAD peptide (purple). n= 10 - 11 (CTL), n= 9 (U18), n= 11 (CCAD) and n= 11 (CCAD + U18) rat neurons and n= 35 - 36 (CTL), n= 27 (U18), n= 33 - 35 (CCAD) and n= 34 (CCAD + U18) dendrites were analyzed across 2 independent isolations. **B**) *Left*, representative super-resolution Airyscan images taken at a focal plane near the PM of CTL (black), U18 (red) or Torin-1-treated (blue) neurons. *Right*, quantification of $Ca_V1.2$ – $K_V2.1$ clustering in the soma (top, yellow) and dendrite (bottom, orange) regions of CTL (black), U18 (red) or Torin-1-treated (blue) neurons. N= 21 (CTL), n = 17 (U18), n = 20 (Torin-1) and n = 22 (Torin-1 + U18) neurons and n = 42 (CTL), n = 33 (U18), n = 38 (Torin-1) and n = 44 (Torin-1 + U18) dendrites were analyzed across 1 isolation. All error bars represent SEM. Statistical significance was calculated using two-way ANOVA. ns: not significant. ns: not significant; **P* < 0.05; ***P* < 0.01; ****P* < 0.001; *****P* < 0.0001. $^{\#\#}P < 0.01$; $^{\#\#\#\#}P < 0.0001$. $^{\#}$ Indicates comparison with the CTL condition. CTL is control, SCRBL is scramble, U18 is U18666A, and CCAD is calcium channel association domain.

Figure S6

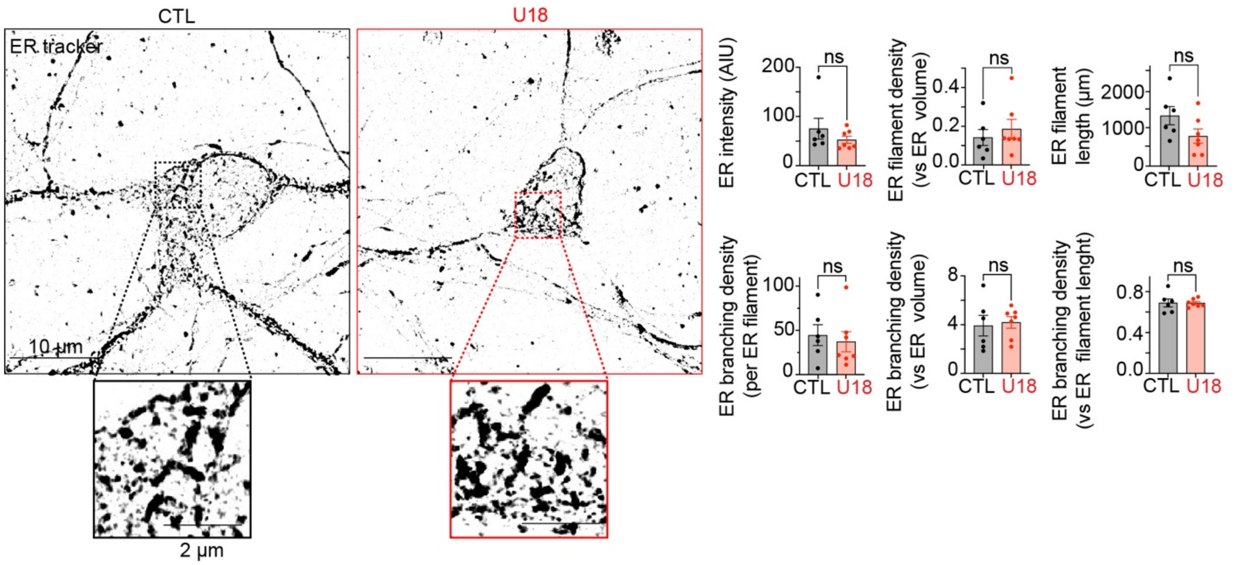

Figure S6. Gross ER morphology is not affected by NPC1 inhibition.

Left, representative maximum intensity projections of the ER from CTL (black) and U18-treated neurons stained with an ER-tracker. *Right*, quantification of ER mean intensity, ER filament density, ER filament length and ER branching density of CTL (black) and U18-treated (red) neurons. N = 6 (CTL) and n = 8 (U18) neurons were analyzed across 1 isolation. Statistical significance was calculated using Mann-Whitney tests (two-tail). ns: not significant. CTL is control and U18 is U18666A.

Figure S7

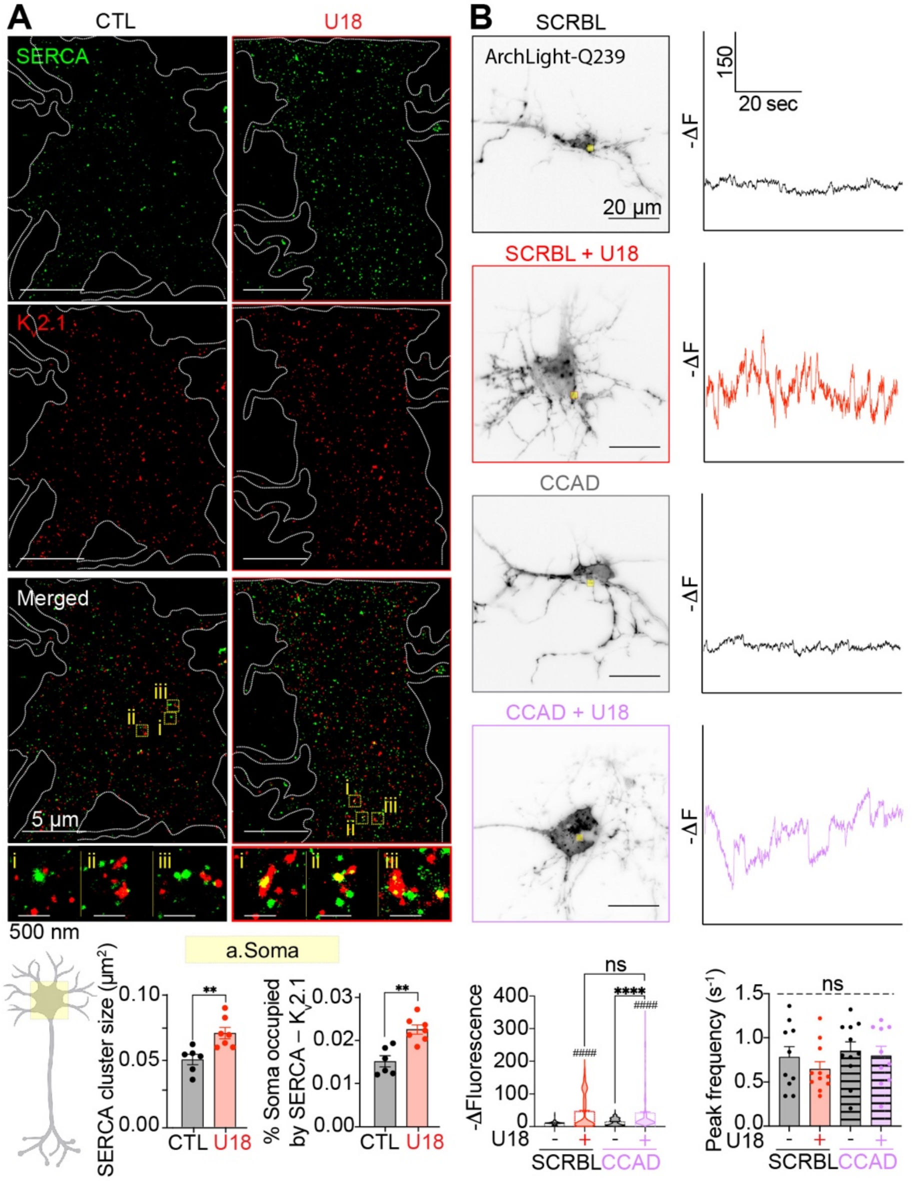

Figure S7. NPC1 dysfunction enhances SERCA–K_V2.1 clustering and PM excitability.

A) *Top*, representative super-resolution TIRF images of CTL (black) and U18-treated (red) neurons co-immunolabeled for SERCA (green) and K_V2.1 (red). *Bottom*, quantification of SERCA cluster size and % soma occupied by SERCA–K_V2.1 of CTL (black) and U18 (red) neurons. n= 6 (CTL) and n= 7 (U18) neurons across one isolation.

B) *Top left*, representative TIRF images of Arch-light Q239 transfected CTL (black), U18-

treated (red) neurons co-incubated with the CCAD peptide (purple). *Top right*, representative ArcLight-Q239 traces of CTL (black) and U18-treated (red) neurons co-incubated with the CCAD peptide (purple). Bottom, quantification of ArcLight-Q239 inverted Δ Fluorescence and peak frequency in CTL (black), U18-treated (red) neurons co-incubated with the CCAD peptide (purple). N = 11 (SCRBL), n = 10 (SCRBL + U18), n = 10 (CCAD) and n = 11 (CCAD + U18) neurons and n = 455 (SCRBL), n = 384 (SCRBL + U18), n = 497 (CCAD) and n = 413 (CCAD + U18) peaks were analyzed across one isolation. All error bars represent SEM. Statistical significance was calculated using Mann-Whitney test (two-tail) in A) and two-way ANOVA in B). ns: not significant; **$P < 0.01$; ****$P < 0.0001$. CTL is control, SCRBL is scramble, U18 is U18666A, and CCAD is calcium channel association domain.

Figure S8

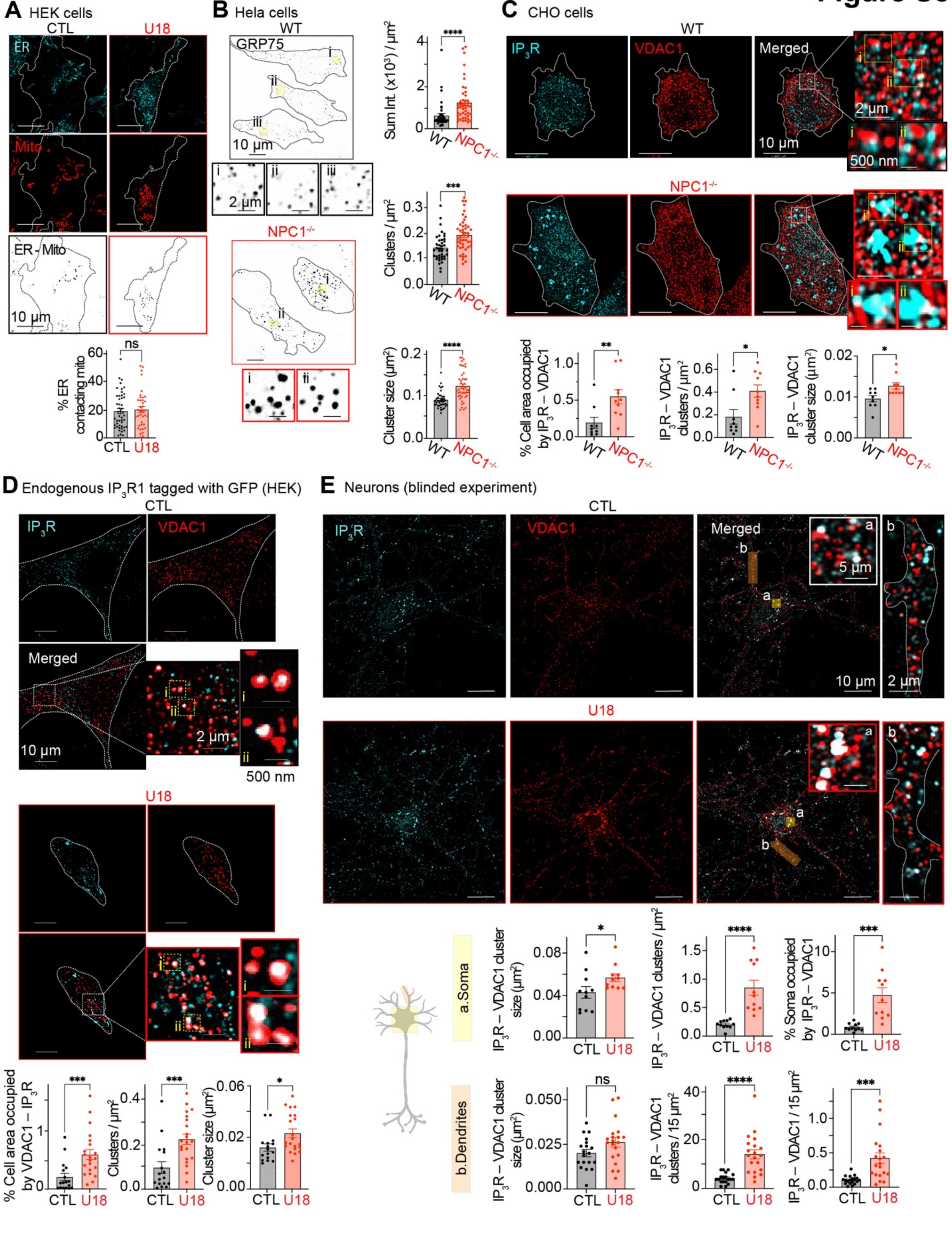

A HEK cells
CTL
U18
ER
Mito
ER - Mito
10 µm
% ER contacting mito
ns
CTL U18
B Hela cells
WT
GRP75
10 µm
2 µm
NPC1-/-
Sum Int. (x10³) / µm²
Clusters / µm²
Cluster size (µm²)
WT NPC1-/-
C CHO cells
WT
IP3R
VDAC1
Merged
2 µm
10 µm
500 nm
NPC1-/-
% Cell area occupied by IP3R – VDAC1
IP3R – VDAC1 clusters / µm²
IP3R – VDAC1 cluster size (µm²)
D Endogenous IP3R1 tagged with GFP (HEK)
CTL
IP3R
VDAC1
Merged
10 µm
2 µm
500 nm
U18
% Cell area occupied by VDAC1 – IP3R
Clusters / µm²
Cluster size (µm²)
CTL U18
E Neurons (blinded experiment)
CTL
IP3R
VDAC1
Merged
5 µm
10 µm
2 µm
U18
a.Soma
IP3R – VDAC1 cluster size (µm²)
IP3R – VDAC1 clusters / µm²
% Soma occupied by IP3R – VDAC1
b.Dendrites
IP3R – VDAC1 clusters / 15 µm²
IP3R – VDAC1 / 15 µm²
CTL U18

Figure S8. NPC1 modulates the nano-distribution of ER - mitochondrial junctions without altering its gross structure.

A) *Top*, representative images taken from CTL (black) and U18-treated (red) HEK cells co-stained with an ER and mitochondrial tracker. *Bottom*, quantification of overlap between ER and mitochondria in CTL (black) and U18-treated (red) cells. N= 48 (CTL) and n= 38 (U18) cells were analyzed across 4 independent experiments. **B**) *Left*, representative images taken from WT (black) and NPC1 knock out (red) HeLa cells immunolabeled for GRP75. Right, quantification of GRP75 total intensity, cluster density and cluster size in WT (black) and NPC1 knock out (red) cells. N= 40 (WT) and 46 (NPC1 knock out) cells were analyzed across 2 independent experiments. **C**) *Top*, representative images from WT (black) and NPC1 knock out (red) HeLa cells co- immunolabeled for IP_3R and VDAC1 across one experiment. *Bottom*, quantification of % cell area occupied by IP_3R-VDAC1 and IP_3R-VDAC1 clustering in WT (black) and NPC1 knock out (red) cells. N = 9 (WT) and n = 10 (NPC1 knock out) cells were analyzed. **D**) *Top*, representative images taken in an internal plane of CTL (black) and U18-treated (red) IP_3R-endogenously expressing HEK cells. Bottom, quantification of % of the cell area occupied by IP_3R-VDAC1 and IP_3R-VDAC1 clustering in CTL (black) and U18-treated (red) IP_3R-endogenously expressing cells. N = 16 - 17 (CTL) and n = 21 (U18) cells were analyzed across two independent experiments. **E**) *Top*, representative super-resolution Airyscan images taken at a focal plane near the PM of CTL (black) and U18 (red) neurons co-immunolabeled for IP_3R and VDAC1 (blinded experiment). *Bottom*, quantification of IP_3R-VDAC1 and IP_3R-VDAC1 clustering in CTL (black) and U18-treated (red) in the soma (top, yellow) and dendrite (bottom, orange) regions of CTL (black) and U18-treated (red) neurons. N= 11 and n= 10 - 11 soma, and n= 19 and n= 20 dendrites were analyzed across one isolation. All error bars represent SEM. Statistical significance was calculated using Mann-Whitney (two-tail) and Unpaired t-tests (two-tail). ns: not significant; *$P < 0.05$; **$P < 0.01$; ***$P < 0.001$; ****$P < 0.0001$. CTL is control and U18 is U18666A.

Figure S9

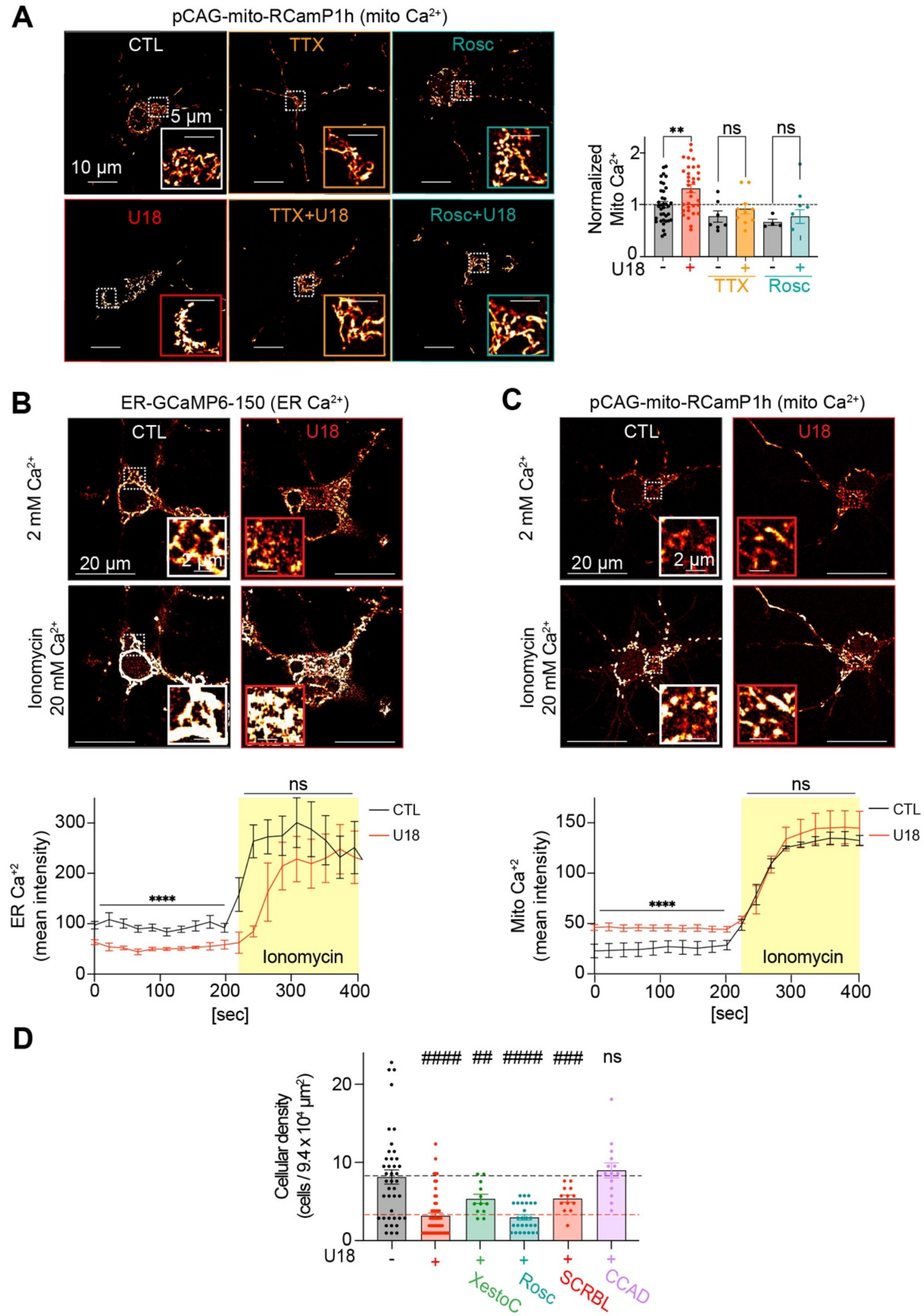

Figure S9. NPC1 regulates mitochondrial Ca^{2+} levels and cellular viability.

A) *Top*, representative super-resolution Airyscan images taken in an internal plane of CTL (black), U18 (red), TTX (orange) and Rosc (cyan) neurons transfected with the Mito-RCamP1h probe. *Bottom,* normalized mitochondrial Ca^{2+} quantification (ratio of before/after adding ionomycin). N= 35 (CTL), n= 33 (U18), n= 7 (TTX), n= 10 (TTX+U18), n= 4 (Rosc) and n= 6 (Rosc+U18) neurons were analyzed across 3 independent isolations. **B**) Top, representative super-resolution Airyscan images taken in an internal plane of CTL (black) and U18 (red) neurons transfected with the Mito-RCamP1h probe. Bottom, representative Mito-RCamP1h raw traces from CTL (black) and U18 (red) neurons before and during ionomycin treatment (in yellow). N= 4 (CTL) and 3 (U18) neurons were analyzed. **C**) Top, representative super-resolution Airyscan images taken in an internal plane of CTL (black) and U18 (red) neurons transfected with the ER-GCamP6-150 probe. Bottom, representative ER-GCamP6-150 raw traces from CTL (black) and U18 (red) neurons before and during ionomycin treatment (in yellow). N= 4 (CTL) and 3 (U18) neurons were analyzed. **D**) Quantification of cellular density of CTL (black), U18 (red), XestoC + U18 (green), Rosc + U18 (cyan), SCRBL +U18 (red) and CCAD + U18 (purple) neurons. N= 41 (DMSO), n= 55 (U18), n= 12 (XestoC + U18), n= 27 (Rosc + U18), n= 13 (SCRBL + U18), n= 14 (CCAD + U18) imaging field areas were imaged and analyzed across 5 independent experiments. All error bars represent SEM. Statistical significance was calculated using the following tests: two-way ANOVA in A), Mann-Whitney (two-tail) test in B-C) and one sample t-test and Wilcoxon test in D). ns: not significant; $^{*}P < 0.05$; $^{**}P < 0.01$; $^{***}P < 0.001$; $^{****}P < 0.0001$. $^{\#}P < 0.05$; $^{\#\#}P < 0.01$; $^{\#\#\#\#}P < 0.0001$. $^{\#}$ Indicates comparison with the CTL condition. CTL is control, U18 is U18666A, CCAD is calcium channel association domain, Rosc is Roscovitine, TTX is tetrodotoxin, Nif is Nifedipine, and XestoC is Xestospongin C.

Figure S10

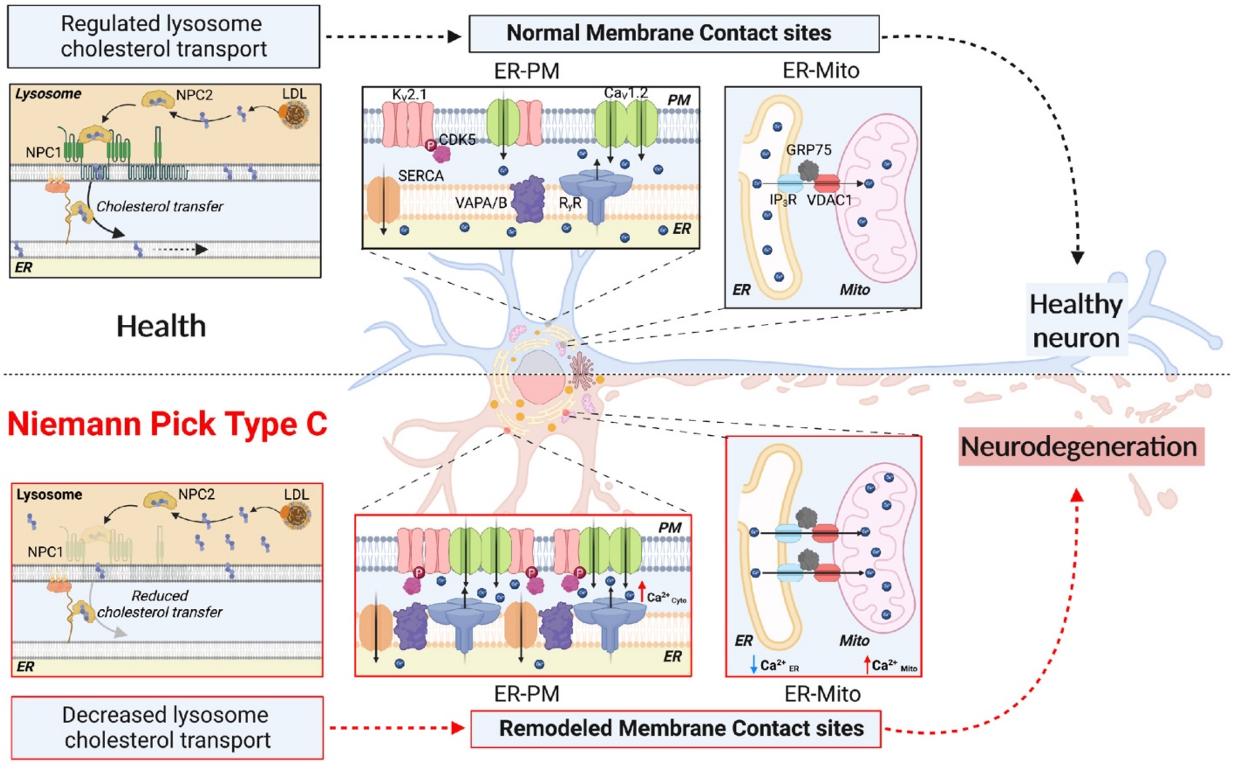

Figure S10. NPC1 loss of function remodels ER–PM and ER–Mito membrane contacts to promote Ca^{2+}_{Mito}-driven neurotoxicity. Graphical abstract showing that NPC1 loss of function increases ER–PM MCSs enriched in SERCA–VAPAB–K_V2.1–Ca_V1.2–RyR and enhances the IP_3R–GRP75–VDAC1 axis, leading to elevations in cytosolic Ca^{2+} and mitochondrial Ca^{2+} driven neurotoxicity.

Supplementary table 1: Resources table

REAGENT or RESOURCE	SOURCE	IDENTIFIER
**Antibodies**		
Mouse anti-K_V2.1 (4 µg/mL for WB and 10 µg/mL for IF)	UC Davis/NIH NeuroMab Facility	K89/34 RRID:AB_2877280
Mouse anti-p(603)K_v2.1 (1:5 for IF)	In house	L61/14 RRID:AB_2315769
Rabbit anti-K_V2.1-KC (1:100 for IF)	In house	RRID: AB_2315767
Rabbit anti-GAPDH (1:1000 for WB)	Proteintech	Cat # 10494-I-AP RRID:AB_2263076
Recombinant mouse anti-Ca_V1.2 (1.8 µg/mL for IF)	In house	N263/31R RRID:AB_2909567
Mouse anti-VAPA/B (10 µg/mL for IF)	UC Davis/NIH NeuroMab Facility	N479/107 RRID:AB_2722711
Goat anti-rabbit IgG (1:1000 for WB)	LI-COR biosciences	Cat # P/N 926-68071; RRID:AB_10956166
Mouse anti-HA-488 (1:100)	Invitrogen	Cat # 2618 3-D488
Goat anti-mouse IgG (1:1000 for WB)	LI_COR biosciences	Cat # P/N 925-32210; RRID:AB_2687825

Goat anti-mouse IgG1 (1:1000 for IF)	Invitrogen	Cat # A21124 (568) RRID:AB_2535766
Goat anti-Mouse (1:1000 for IF)	Invitrogen	Cat # A21236 (647) RRID:AB_2535805 Cat # A11031 (568) RRID:AB_144696
Goat anti-Rabbit (1:1000 for IF)	Invitrogen	Cat # A21245 (647) RRID:AB_2535813 Cat # A21429 (555) RRID:AB_2535850
Goat anti-Mouse IgG1 (1:250 for IF)	Sigma-Aldrich	Cat # SAB4600314
Rabbit anti-GRP75 (1:100 for IF)	Abcam	Cat # Ab2799 RRID:AB_303311
Rabbit anti-Ca_v1.2 (1:333 and 1:200 for IF and 1:500 for WB)	Alomone Labs	Cat # ACC-003 RRID:AB_2039771
Mouse anti-VDAC1 (1:100 for IF)	Abcam	Cat # Ab14734 RRID:AB_443084
Rabbit anti-SERCA (10 µg/mL for IF)	Abcam	Cat # Ab2861 RRID:AB_2061425

Rabbit anti-$Ca_v1.3$ (10 µg/mL for IF)	Alomone Labs	Cat # ACC-005<br>PRID:AB_2039775
Rabbit anti-IP_3R (18 µg/mL for IF)	Abcam	Cat # Ab5804<br>RRID:AB_305124
Rabbit anti-$Ca_v2.1$ (1:200 for IF)	Alomone Labs	Cat # ACC-001<br>RRID:AB_2039764
Mouse anti-RyR (1:100 for IF)	Abcam	34C<br>Cat # Ab2868<br>RRID:AB_2183051
Mouse anti-Calbindin (2 µg/mL for IF)	UC Davis/NIH NeuroMab Facility	L109/57<br>RRID: AB_2619740
**Chemicals, Peptides, and Recombinant Proteins**		
Tetrodotoxin (200 nM, O/N)	Sigma-Aldrich	Cat # T8024
U18666A (10 µM, O/N)	Sigma-Aldrich	Cat # 662015
Retigabine (10 µM, O/N)	APExBIO	Cat # A3758
Roscovitine (10 µM, O/N)	Sigma-Aldrich	Cat # R7772
Ionomycin (2.5 µM, 6 minutes)	Sigma-Aldrich	Cat # I9657
Xestospongin C (1 µM, O/N)	Sigma-Aldrich	Cat # X2628
Fillipin (100 µg/mL, 2h)	Millipore Sigma	Cat # F9765
ER Tracker Blue-White DPX (100 nM, 20 min)	Invitrogen	Cat # E12353
MitoTracker Deep Ref FM (50 nM, 20 min)	Invitrogen	Cat # M22426
TAT-HA-C1aB (CCAD,1 µM, 48h)<br>Seq:<br>GRKKRRQRRRYPYDVPDYAHLSPNKWKW	Genscript	N/A

TAT-HA-ClaB-Scr (Scr,1 μM, 48h) Seq: GRKKRRQRRRYPYDVPDYANLKWSHPKW	Genscript	N/A
TAT-FFAT-HA (472-481) (FFAT, 1 μM, 48h) Seq: GRKKRRQRRRPQSEDEFYDALSYPYDVPDYA	Genscript	N/A
TAT-FFAT-HA (472-481)-scr (FFAT-Scr, 1 μM, 48h) Seq: GRKKRRQRRRAYESDQLPSFDEYPYDVPDYA	Genscript	N/A
Fluo-4-AM (2.5 μM, 30min)	Life Technologies	Cat # F14201
B27	Gibco	Cat # 17504-044
Glutamax	Gibco	Cat # 35050-061
Lipofectamine 2000	Invitrogen	Cat # 11668-027
Complete Mini protease inhibitor cocktail	Roche	Cat # 11836170001
SEA BLOCK Blocking Buffer	Thermo Scientific	Cat # 37527
Paraformaldehyde	Electron Microscopy Sciences	Cat # 15710
Lipofectamine LTX	Invitrogen	Cat # 15338-030
**Experimental Models: Cell Lines**		
HeLa cells wild type	Sigma-Aldrich	N/A
HeLa cells $NPC1^{-/-}$	Provided by Dr. Judith Storch (Rutgers)	N/A
tsA 201 cells	Sigma-Aldrich (St Louis, MO, USA)	Cat # 96121229
Mouse pre-natal cortical neurons	C57BL/6 fetuses E13-16 (Jackson Laboratory)	RRID:IMSR_JAX: 000664

Rat pre-natal cortical neurons	Sprague-Dawley fetuses E13-16	RRID:RGD_737891
**Experimental Models: Organisms/Strains**		
Mouse: $NPC1^{I1061T}$: C57BL/6 Strain knock-in of $NPC1^{I1061T}$	Provided by Dr. Daniel Ory. Praggastis *et al.*, 2015.	N/A
Mouse: wild type (WT): C57BL/6	Jackson Laboratory	RRID:IMSR_JAX:000664
Sprague-Dawley rat	Charles River	RRID:RGD_737891
**Recombinant DNA**		
GFP-MAPPER (2 μg/ 650,000 neurons, 48h)	Dr. Jen Liou, UT Southwestern	Chang *et al.*, 2013
ER-GCaMP6-150 (4μg/650,000 neurons, 48h)	Addgene	Cat # 86918
pCAG-mito-RCaMP1h (4μg/650,000 neurons, 48h)	Addgene	Cat # 105013
GCaMP3-$Kv2.1_{P4O4W}$ (1.1μg/650,000 neurons, 48h)	In house	Vierra et al., 2019
SPLICS PM-ER Long P2A (1μg/650,000 neurons, 48h)	Addgene	Cat # 164112
SPLICS PM-ER Short P2A (1μg/650,000 neurons, 48h)	Addgene	Cat # 164111
**Software and Algorithms**		
Image J	ImageJ	RRID:SCR_003070
Prism	GraphPad	RRID:SCR_002798
Microsoft Excel	Microsoft	RRID:SCR_016137
ClampFit	Molecular Devices	N/A

IMARIS	Oxford Instruments	N/A
Micro-manager	NIH	https://micro-manager.org
ZEN imaging software	Zeiss	RRID: SCR_013672
**Commercial Kits**		
Live/Dead Cell Viability Assay Kit (for Mammalian cells-24 well)	Bio Vision	Cat # K502-100
Papain Dissociation System	Worthington	Cat # LK003150
Duolink In Situ PLA probe Anti-Mouse MINUS	Sigma-Aldrich	Cat # DUO92004-100RXN
Duolink In Situ PLA Probe Anti-Rabbit PLUS	Sigma-Aldrich	Cat # DUO92002-100RXN
H2DCFDA	Invitrogen	Cat # D399
MitoProbe JC-1	Abcam	Cat # ab113850
Pierce BCA Protein Assay Kit	Thermo Fisher	Cat # 23225

Uncropped WB membranes for Fig 5

- Representative cropped images in the article are shown in black dashed rectangles.
- Some membranes were cut in order to blot different proteins simultaneously.
- Some membranes were imaged in specific regions of interest to increase image resolution.
- Ponceau staining was performed in all membranes to ensure proper protein transfer.

Fig 5.C - p35 and p39

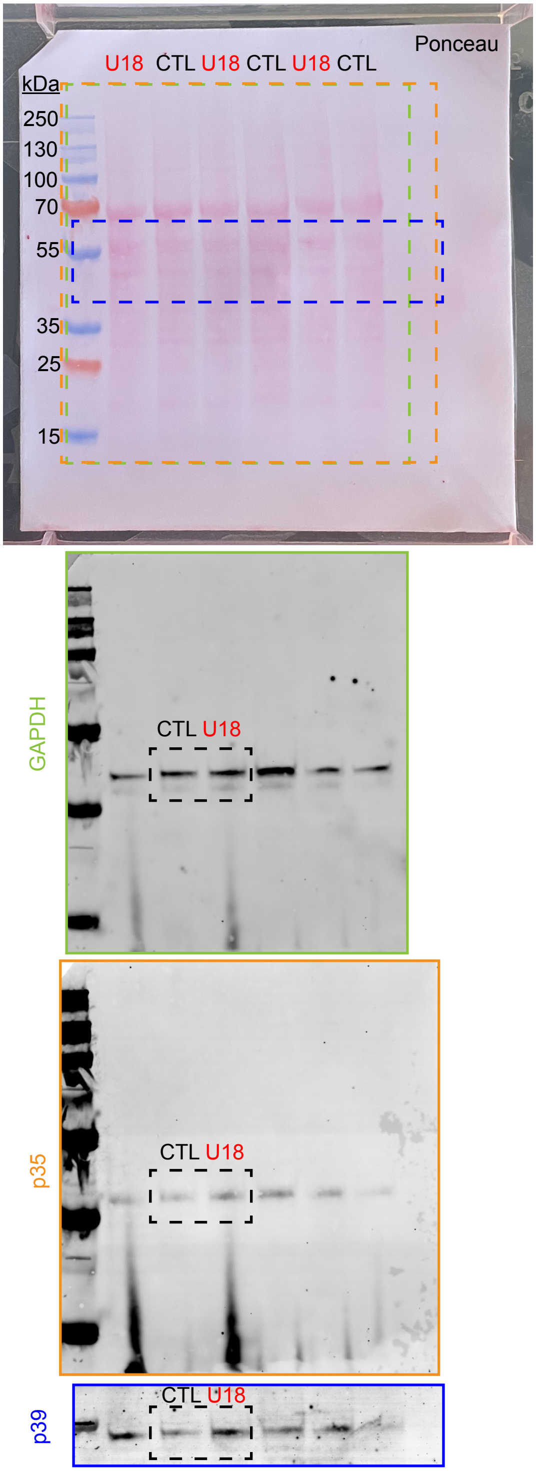

Fig 5.C - CDK5

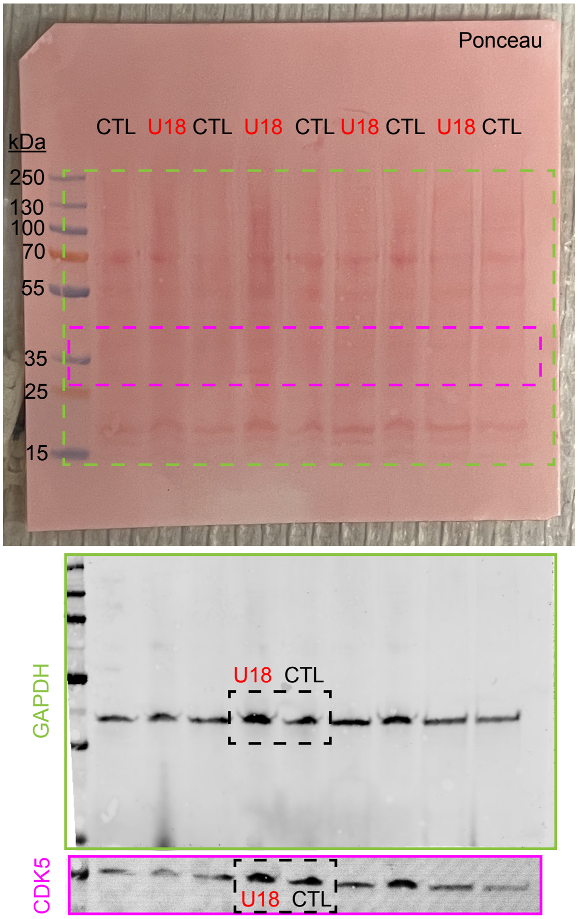

Uncropped WB membranes for Fig S3

- Representative cropped images in the article are shown in black dashed rectangles.
- Gel on the left was used for Ca_V1.2 and K_V2.1 quantification.
- One CTL and one U18 sample were loaded again in a separate gel in order to get better K_V2.1 representative images (on the right).
- Some membranes were cut in order to blot different proteins simultaneously.
- Some membranes were imaged in specific regions of interest to increase image resolution.
- Ponceau staining was performed in all membranes to ensure proper protein transfer.

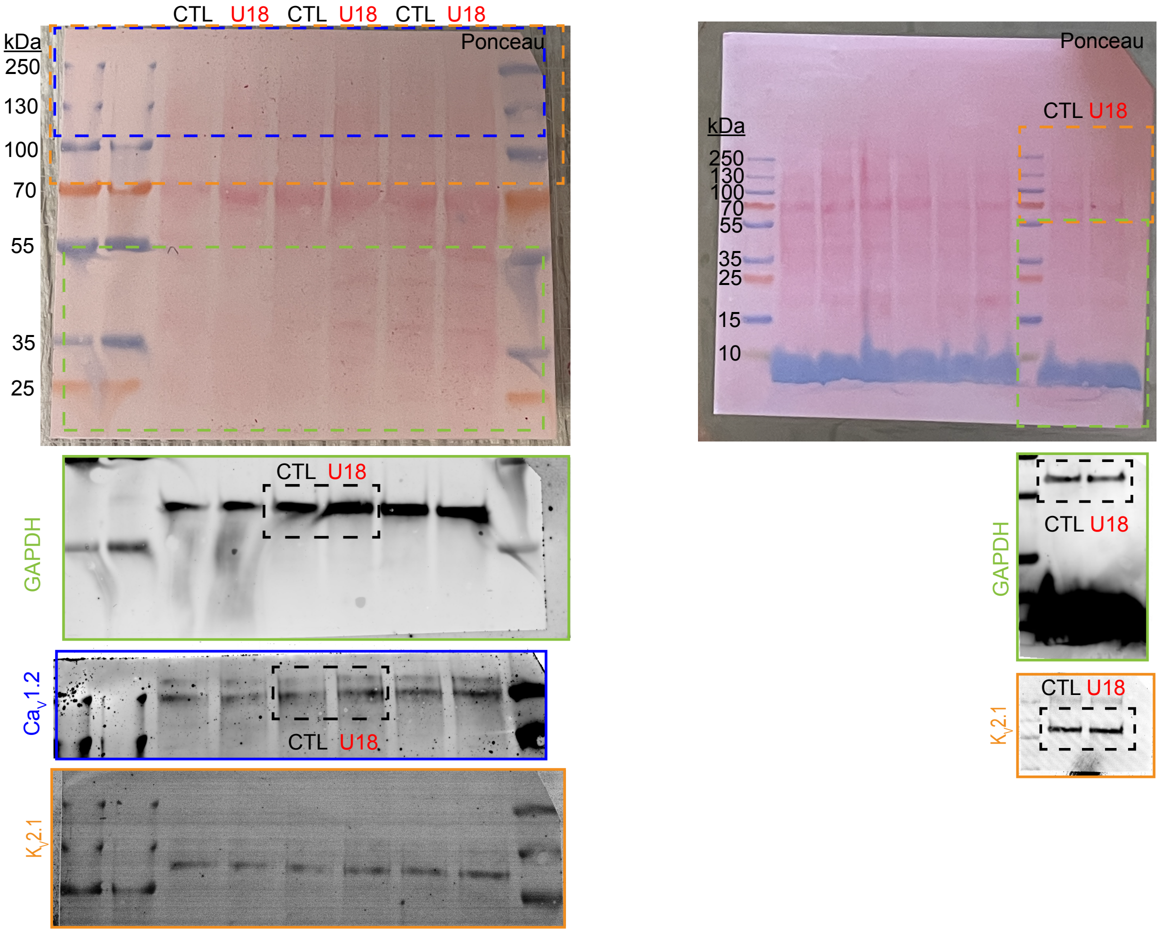

Supplement: Supplementary file 1 — Supplementary information [file 41467_2023_39937_MOESM1_ESM.pdf]
