# Supplementary material for: NPC1-dependent alterations in KV2.1–CaV1.2 nanodomains drive neuronal death in models of Niemann-Pick Type C disease
Source: Nat Commun. 2023 Jul 28;14:4553. doi: 10.1038/s41467-023-39937-w (PMC10382591; doi:10.1038/s41467-023-39937-w)
Supplement: Supplementary file 3 — Description of Additional Supplementary Files [file 41467_2023_39937_MOESM3_ESM.pdf]

Description of Additional Supplementary Files

File name: Supplementary Movie 1

Description: Elevated $Ca_V1.2$–$K_V2.1$ colocalization in $NPC1^{I1061T}$ Purkinje neurons. Representative 3D videos of $Ca_V1.2$ (in green) and $K_V2.1$ (in pink) masks created in the soma region of WT and $NPC1^{I1061T}$ Purkinje neurons immunolabeled for $Ca_V1.2$ and $K_V2.1$.

File name: Supplementary Movie 2

Description: NPC1-dependent increased Ca^{2+} activity at $K_V2.1$-associated domains is mediated by $Ca_V1.2$–$K_V2.1$–VAPA/B. Representative videos of cortical neurons at DIV 14-16 transfected with the GCaMP3-$K_v2.1_{P4O4W}$ construct under CTL, U18, CCAD + U18 and FFAT + U18 conditions.
