# Supplementary material for: NPC1-dependent alterations in KV2.1–CaV1.2 nanodomains drive neuronal death in models of Niemann-Pick Type C disease
Source: Nat Commun. 2023 Jul 28;14:4553. doi: 10.1038/s41467-023-39937-w (PMC10382591; doi:10.1038/s41467-023-39937-w)
Supplement: Supplementary file 6 — Reporting Summary [file 41467_2023_39937_MOESM6_ESM.pdf]

nature portfolio

Corresponding author(s): Eamonn James Dickson

Last updated by author(s): Apr 21, 2023

Reporting Summary

Nature Portfolio wishes to improve the reproducibility of the work that we publish. This form provides structure for consistency and transparency in reporting. For further information on Nature Portfolio policies, see our Editorial Policies and the Editorial Policy Checklist.

Statistics

For all statistical analyses, confirm that the following items are present in the figure legend, table legend, main text, or Methods section.

n/a	Confirmed	
☐	☒	The exact sample size (*n*) for each experimental group/condition, given as a discrete number and unit of measurement
☐	☒	A statement on whether measurements were taken from distinct samples or whether the same sample was measured repeatedly
☐	☒	The statistical test(s) used AND whether they are one- or two-sided<br>*Only common tests should be described solely by name; describe more complex techniques in the Methods section.*
☐	☒	A description of all covariates tested
☐	☐	A description of any assumptions or corrections, such as tests of normality and adjustment for multiple comparisons
☐	☒	A full description of the statistical parameters including central tendency (e.g. means) or other basic estimates (e.g. regression coefficient) AND variation (e.g. standard deviation) or associated estimates of uncertainty (e.g. confidence intervals)
☐	☒	For null hypothesis testing, the test statistic (e.g. *F*, *t*, *r*) with confidence intervals, effect sizes, degrees of freedom and *P* value noted<br>*Give P values as exact values whenever suitable.*
☐	☒	For Bayesian analysis, information on the choice of priors and Markov chain Monte Carlo settings
☐	☒	For hierarchical and complex designs, identification of the appropriate level for tests and full reporting of outcomes
☒	☐	Estimates of effect sizes (e.g. Cohen's *d*, Pearson's *r*), indicating how they were calculated

Our web collection on statistics for biologists contains articles on many of the points above.

Software and code

Policy information about availability of computer code

Data collection	Commercially available software used in this study:<br>Zeiss AiryScan 880: Zeiss Zen (Zeiss Zen v2.3 SP1)<br>Single molecule localization microscopy - Leica LAS X (3.7.2.22383)<br>Micromanager (1.4.21).<br>Biochemistry data was imaged using a Azure Biosystems Sapphire Biomolecular Imager.
Data analysis	All imaging was analyzed with ImageJ/FIJI (NIH, version V2.1.0/1.53h) or IMARIS (version 10); quantification of protein expression on western blots was performed using ImageJ/FIJI and Adobe Photoshop (Creative Suite version 2022); all graphing and statistics were performed in Graphpad Prism (version 9 for Mac; GraphPad Software, San Diego, California USA).

For manuscripts utilizing custom algorithms or software that are central to the research but not yet described in published literature, software must be made available to editors and reviewers. We strongly encourage code deposition in a community repository (e.g. GitHub). See the Nature Portfolio guidelines for submitting code & software for further information.

Data

Policy information about availability of data

All manuscripts must include a data availability statement. This statement should provide the following information, where applicable:

- Accession codes, unique identifiers, or web links for publicly available datasets
- A description of any restrictions on data availability
- For clinical datasets or third party data, please ensure that the statement adheres to our policy

All datasets generated and/or analyzed during this study are available from the corresponding author upon reasonable request

Research involving human participants, their data, or biological material

Policy information about studies with human participants or human data. See also policy information about sex, gender (identity/presentation), and sexual orientation and race, ethnicity and racism.

Reporting on sex and gender	N/A
Reporting on race, ethnicity, or other socially relevant groupings	N/A
Population characteristics	N/A
Recruitment	N/A
Ethics oversight	N/A

Note that full information on the approval of the study protocol must also be provided in the manuscript.

Field-specific reporting

Please select the one below that is the best fit for your research. If you are not sure, read the appropriate sections before making your selection.

☒ Life sciences ☐ Behavioural & social sciences ☐ Ecological, evolutionary & environmental sciences

For a reference copy of the document with all sections, see nature.com/documents/nr-reporting-summary-flat.pdf

Life sciences study design

All studies must disclose on these points even when the disclosure is negative.

Sample size	The number of replicates is based on previously published observations to reached statistical differences between datasets. See methods section for references.
Data exclusions	No data was excluded from datasets
Replication	Key experiments were conducted by multiple investigators across separate days and repeated from at least two isolations. All attempts at replication were successful. Specific number of independent replicates is noted in the figure legends.
Randomization	Isolations were conducted across multiple days and by multiple people. Experiments we conducted in random order (i.e. control expeirment imaged first and then treatment group, or vice versa).
Blinding	Key experiments (noted in the manuscript) were subjected to blinding.

Reporting for specific materials, systems and methods

We require information from authors about some types of materials, experimental systems and methods used in many studies. Here, indicate whether each material, system or method listed is relevant to your study. If you are not sure if a list item applies to your research, read the appropriate section before selecting a response.

Materials & experimental systems

n/a	Involved in the study	
☐	☒	Antibodies
☐	☒	Eukaryotic cell lines
☒	☐	Palaeontology and archaeology
☐	☒	Animals and other organisms
☒	☐	Clinical data
☒	☐	Dual use research of concern
☒	☐	Plants

Methods

n/a	Involved in the study	
☒	☐	ChIP-seq
☒	☐	Flow cytometry
☒	☐	MRI-based neuroimaging

Antibodies

Antibodies used

All antibodies are commercially available or upon request by source, and the applications have been tested by the manufacturer and by us in different studies. Antibodies specifically tested by us include:
Mouse anti-KV2.1(4 µg/mL for WB and 10 µg/mL for IF); UC Davis/NIH NeuroMab Facility K89/34; RRID:AB_2877280
Mouse anti-p(603)Kv2.1(1:5 for IF); In house; L61/14; RRID:AB_2315769
Rabbit anti-KV2.1-KC (1:100 for IF); In house; RRID: AB_2315767
Rabbit anti-GAPDH (1:1000 for WB); Proteintech Cat # 10494-I-AP; RRID:AB_2263076
Recombinant mouse anti-CaV1.2; (1.8 µg/mL for IF); In house N263/31R; RRID:AB_2909567
Mouse anti-VAPA/B (10 µg/mL for IF); UC Davis/NIH NeuroMab Facility; N479/107 RRID:AB_2722711
Goat anti-rabbit IgG (1:1000 for WB); LI-COR biosciences; Cat # P/N 926-68071; RRID:AB_10956166
Mouse anti-HA-488 (1:100); Invitrogen Cat # 2618 3-D488
Goat anti-mouse IgG (1:1000 for WB); LI_COR biosciences Cat # P/N 925-32210; RRID:AB_2687825
Goat anti-mouse IgG1 (1:1000 for IF); Invitrogen Cat # A21124 (568);RRID:AB_2535766
Goat anti-Mouse (1:1000 for IF); Invitrogen Cat # A21236 (647); RRID:AB_2535805
Cat # A11031(568); RRID:AB_144696
Goat anti-Rabbit (1:1000 for IF); Invitrogen Cat #A21245 (647); RRID:AB_2535813
Cat # A21429 (555); RRID:AB_2535850
Goat anti-Mouse IgG1(1:250 for IF); Sigma-Aldrich Cat # SAB4600314
Rabbit anti-GRP75 (1:100 for IF); Abcam Cat # Ab2799; RRID:AB_303311
Rabbit anti-Cav1.2 (1:333 and 1:200 for IF and 1:500 for WB); Alomone Labs Cat # ACC-003; RRID:AB_2039771 Mouse anti-VDAC1 (1:100 for IF); Abcam Cat # Ab14734; RRID:AB_443084
Rabbit anti-SERCA (10 µg/mL for IF); Abcam Cat # Ab2861; RRID:AB_2061425
Rabbit anti-Cav1.3 (10 µg/mL for IF); Alomone Labs Cat # ACC-005; PRID:AB_2039775
Rabbit anti-IP3R (18 µg/mL for IF); Abcam Cat # Ab5804; RRID:AB_305124
Rabbit anti-Cav2.1(1:200 for IF); Alomone Labs Cat # ACC-001; RRID:AB_2039764
Mouse anti-RyR (1:100 for IF); Abcam 34C; Cat # Ab2868; RRID:AB_2183051
Mouse anti-Calbindin (2 µg/mL for IF); UC Davis/NIH NeuroMab Facility; L109/57;RRID: AB_2619740

Validation

Abbreviations: IF (immuno fluorescence), IB (immunoblot), IHC (immunohistochemistry), KO (knock out), Western blot (WB), immunoprecipitation (IP)
Mouse anti-KV2.1: RRID:AB_2877280: Passed IF, IB, IHC, KO validations
Mouse anti-p(603)Kv2.1(1:5 for IF): Passed IF, IB, IHC, KO validations
Rabbit anti-KV2.1-KC (1:100 for IF): Passed IF, IB, IHC, KO validations
Rabbit anti-GAPDH (1:1000 for WB);RRID:AB_2263076: Passed WB, IP, IHC, IP, and FC validations.
Recombinant mouse anti-CaV1.2; (1.8 µg/mL for IF);N263/31R; RRID:AB_2909567; assed IF, IB, IHC, WB validations.
Mouse anti-VAPA/B (10 µg/mL for IF); RRID:AB_2722711; Passed IM, IHC, IF validations.
Goat anti-rabbit IgG (1:1000 for WB); LI-COR biosciences; RRID:AB_10956166; Passed WB validations.
Mouse anti-HA-488 (1:100); Invitrogen Cat # 2618 3-D488; Passed IF validations.
Goat anti-mouse IgG (1:1000 for WB); Cat # P/N 925-32210; RRID:AB_2687825; Passed WB validations.
Goat anti-mouse IgG1 (1:1000 for IF); Invitrogen Cat # A21124 (568);RRID:AB_2535766; Passed WB validations.
Goat anti-Mouse (1:1000 for IF); Invitrogen Cat # A21236 (647); RRID:AB_2535805; Passed IF validations.
Cat # A11031(568); RRID:AB_144696
Goat anti-Rabbit (1:1000 for IF); Invitrogen Cat #A21245 (647); RRID:AB_2535813; Passed IF validations.
Cat # A21429 (555); RRID:AB_2535850
Goat anti-Mouse IgG1(1:250 for IF); Sigma-Aldrich Cat # SAB4600314. Passed WB validations.
Rabbit anti-GRP75 (1:100 for IF); Abcam Cat # Ab2799; RRID:AB_303311; Passed IF and KO validations.
Rabbit anti-Cav1.2 (1:333 and 1:200 for IF and 1:500 for WB); Alomone Labs Cat # ACC-003; RRID:AB_2039771: Passed WB validations. Confirmed by amino acid analysis and mass spectrometry.
Mouse anti-VDAC1(1:100 for IF); Abcam Cat # Ab14734; RRID:AB_443084; Passed IF and WB validations.
Rabbit anti-SERCA (10 µg/mL for IF); Abcam Cat # Ab2861; RRID:AB_2061425; Passed IF and WB validations
Rabbit anti-Cav1.3 (10 µg/mL for IF); Alomone Labs Cat # ACC-005; PRID:AB_2039775; Passed KO valdiations.
Rabbit anti-IP3R (18 µg/mL for IF); Abcam Cat # Ab5804; RRID:AB_305124; validated by WB, IF, KO.
Rabbit anti-Cav2.1(1:200 for IF); Alomone Labs Cat # ACC-001; RRID:AB_2039764; Passed IF and WB validations
Mouse anti-RyR (1:100 for IF); Abcam 34C; Cat # Ab2868; RRID:AB_2183051; Passed IF and WB validations

Mouse anti-Calbindin (2 µg/mL for IF); UC Davis/NIH NeuroMab Facility; L109/57;RRID: AB_2619740; Passed IF and WB validations

Eukaryotic cell lines

Policy information about cell lines and Sex and Gender in Research

Cell line source(s)	tsA201 cells were purchased from Sigma (Cat # 96121229), CHO WT (ATCC (CRL-9618) and NPC1-/- cells were a kind gift from Dr. Ory (Washington University, St. Louis, MO), HeLa WT (ATTC #CCL-2) and NPC1-/- cells were a kind gift from Dr. Judith Storch (Rutgers)
Authentication	tsA cell lines and HeLa cells were authenticated by STR profiling. NPC1-/- Knockout cell lines were authenticated by western blot
Mycoplasma contamination	cell lines were regularly tested for mycoplasm contamination and were not contaminated
Commonly misidentified lines (See ICLAC register)	No commonly misidentified cell lines were used in this study.

Animals and other research organisms

Policy information about studies involving animals; ARRIVE guidelines recommended for reporting animal research, and Sex and Gender in Research

Laboratory animals	Embryonic E18 C57/B6 Male and Female animals were used to generate neuronal cultures. 60 Day old WT and NPC111061T C57/B6 Male and Female animals were used to generate brain sections for immunolabeling. Mice were housed in a 12-hr dark-light cycle and experiments were performed during the light phase. Mice were conventionally housed with free access to food and water ad libitum.
Wild animals	The study did not involve wild animals
Reporting on sex	Cortical neurons from mice of both sexes were dissociated at embryonic day 15-18 (E15-18).For each neuronal dataset experiments were performed from 1-4 independent neuronal cultures with each isolation containing 6-8 pups.
Field-collected samples	The study did not involve any field-collected samples.
Ethics oversight	Animals studies were approved and overseen by the University of California Davis Animal Care and Use Committee (protocol 20974).

Note that full information on the approval of the study protocol must also be provided in the manuscript.
